# Cell-Based Therapy Manufacturing in Stirred Suspension Bioreactor: Thoughts for cGMP Compliance

**DOI:** 10.3389/fbioe.2020.599674

**Published:** 2020-11-26

**Authors:** Suman C. Nath, Lane Harper, Derrick E. Rancourt

**Affiliations:** ^1^Department of Biochemistry & Molecular Biology, Cumming School of Medicine, University of Calgary, Calgary, AB, Canada; ^2^McCaig Institute for Bone and Joint Health, Cumming School of Medicine, University of Calgary, Calgary, AB, Canada

**Keywords:** cell-based therapy, biologics manufacturing, cGMP, genetic engineering, integrated bioprocessing, bioreactor

## Abstract

Cell-based therapy (CBT) is attracting much attention to treat incurable diseases. In recent years, several clinical trials have been conducted using human pluripotent stem cells (hPSCs), and other potential therapeutic cells. Various private- and government-funded organizations are investing in finding permanent cures for diseases that are difficult or expensive to treat over a lifespan, such as age-related macular degeneration, Parkinson’s disease, or diabetes, etc. Clinical-grade cell manufacturing requiring current good manufacturing practices (cGMP) has therefore become an important issue to make safe and effective CBT products. Current cell production practices are adopted from conventional antibody or protein production in the pharmaceutical industry, wherein cells are used as a vector to produce the desired products. With CBT, however, the “cells are the final products” and sensitive to physico- chemical parameters and storage conditions anywhere between isolation and patient administration. In addition, the manufacturing of cellular products involves multi-stage processing, including cell isolation, genetic modification, PSC derivation, expansion, differentiation, purification, characterization, cryopreservation, etc. Posing a high risk of product contamination, these can be time- and cost- prohibitive due to maintenance of cGMP. The growing demand of CBT needs integrated manufacturing systems that can provide a more simple and cost-effective platform. Here, we discuss the current methods and limitations of CBT, based upon experience with biologics production. We review current cell manufacturing integration, automation and provide an overview of some important considerations and best cGMP practices. Finally, we propose how multi-stage cell processing can be integrated into a single bioreactor, in order to develop streamlined cGMP-compliant cell processing systems.

## Introduction

Human pluripotent stem cells (hPSCs), including embryonic stem cells (ESCs) (Thomson et al., 1998) and induced pluripotent stem cells (iPSCs) (Takahashi et al., 2007) are attractive tools in the field of regenerative medicine because of their ability to self-renew and differentiate into any cell type in the human body. Use of these cells increased exponentially after the discovery of hiPSCs in 2007 (Guhr et al., 2018). Recently, hundreds of biotechnology companies were founded with the mission to treat degenerative diseases using these cells. Age-related macular degeneration (AMD), type I diabetes mellitus, heart failure, Parkinson’s disease and spinal cord injury are the most common degenerative diseases being treated with hPSCs (Trounson and McDonald, 2015).

Although hiPSCs are a better source for autologous CBT, they are less preferable for clinical trials because they are less genetically stable than hESCs (Attwood and Edel, 2019). Viral vectors using for iPSC reprogramming integrate into the genome and poses risk of insertional mutagenesis (Baum, 2007). Moreover, genetic modification can cause mutations that associated with cancer (Gore et al., 2011). Evidence of transgene reactivation after iPSC reprogramming also poses risk after transplantation (Galat et al., 2016).

Some clinical studies have already begun using hiPSCs derived from patients. A clinical trial for the treatment of wet AMD has recently been conducted by the Masayo Takahashi group from the Riken Center for Developmental Biology (Reardon and Cyranoski, 2014). Similarly, Jun Takahashi from Kyoto University is also conducting a clinical trial using hiPSCs to treat Parkinson’s disease (Cyranoski, 2018). There are also several clinical trials in the United States using hiPSCs for the treatment of various diseases such as β-thalassemia, liver disease, diabetes, etc. and their use is increasing worldwide (Kimbrel and Lanza, 2015).

Since stem CBT trials are proliferating, many clinical studies continue to use both hESCs and hiPSCs. About 8141 CBTs and 1657 stem CBTs were found based upon searches recently performed on clinicaltrials.gov (October, 2020) (ClinicalTrials.gov, 2020). However, as speculated from previous clinical studies, the percentage of success is quiet low. Of the 315 clinical trials carried out, only 0.3% went to Phase 4 (Trounson and McDonald, 2015). The low percentage of clinical trial completion depends on different factors. One of the main factors is the design and implementation of cost-effective, high safety production practices required by regulatory bodies. In addition, multi-dose production costs also hamper the success rate of clinical trials. Since the global revenues from CBT in 2018 were approximately a billion dollars and are forecasted to be in the tens of billions by 2025, a great deal of attention is needed to produce high-quality cells to treat incurable diseases (Davie et al., 2012; PR Newswire, 2019).

The production of stem cell-derived biologics is adapted from the production of conventional pharmaceutical proteins and vaccines. The production of conventional biologics involves the following basic steps: Isolation and identification of raw materials, formulation, filling, packaging and storage, where total processing stops when final products are stored. This provides a very basic model as the production of conventional biologics. Yet CBT products differ in various significant ways. In biologics, cells are used as a platform for the production of desired therapeutic proteins. Cells are discarded after a batch and new cells with the requisite protein expression are used to produce the next batch. Proteins produced in this way are generally stable, uniform, and easily characterized, varying little between batches.

**FIGURE 1:**
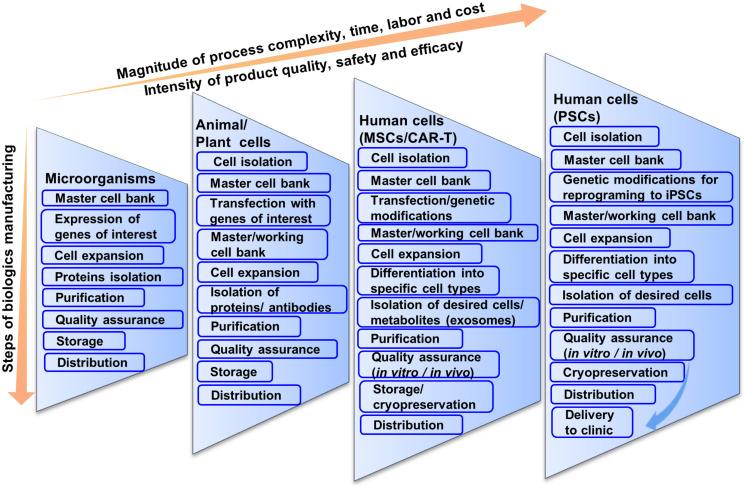
Schematic illustration of current multi-step cell manufacturing strategies in planar culture for cell therapy applications.

In cell-based therapies, the final products are cells that are sensitive to the physical or chemical attributes of the resident environment and are prone to spontaneous change. Therefore, these considerations must be taken into account when translating from bench to clinic (Roh et al., 2016). The need for CBT products are emerging from various cell lines including chimeric antigen receptor T-cell (CAR-T), retinal pigment epithelial cell, neural cell, hepatic cell, cardiac cell, mesenchymal stem cell (MSCs), ESCs, iPSCs, etc. for treating various degenerative diseases. In this review, we will focus on cell therapy development ranging from unipotent to pluripotent cells, namely, CAR-T cells (unipotent cells), MSCs (multipotent) and iPSCs (pluripotent) (Figure 1).

Since CBT needs much consideration for producing large cell numbers, using conventional cell processing systems makes it more complicated and sometimes impossible. Being a multi-step process that can cause batch variability, inefficiency and low quality of transplantable cells in plate culture, there are too many possibilities for human error. CBT needs to be simplified and made more direct. In this context, we will discuss some of the current limitations of cell production strategies. We will propose how to possibly overcome these limitations by integrating the entire process into a single bioreactor system due to advantages over plate culture (Table 1). We will also discuss how genetic modification- transfection or transduction, reprogramming, differentiation, purification and development of final products in a single bioreactor can be integrated. In this context, we also discuss some basic, high level cGMP considerations for CBT biomanufacturing.

**TABLE 1:** Comparison of pros and cons of adherent and bioreactor culture.

Cultures	Pros	Cons
**Adherent**	• Easy to handling • Easy for cell visualization • Easy for genetic manipulation	• Low final cell density • High medium consumption • Limited growth surface area • Large numbers of culture vessels are required • Online sampling is not possible • Labor intensive • Low scalability • Difficulties in cell harvesting (need to detach from culture surface) • Difficult for automation because of cell detachment step
**Bioreactor**	• High final cell density • Low medium consumption • Unlimited growth surface area • Single vessel is enough • Online sampling • Flexible labor • High scalability • Easy for harvesting (ready to use as aggregate) • Suitable for automation	• Handling requires expertise • Cell visualization requires an extra step • Difficulties in genetic manipulation

## Conventional Practices for CBT Product Manufacturing

As stated earlier, cell processing differs widely from pharmaceutical proteins or vaccine production although current manufacturing practices are based on conventional biologics manufacturing that may not be compatible for CBT (Bennett, 2018; Sterlling, 2018). For example, in biologics manufacturing, microorganisms containing genes of interests are expanded and the proteins are isolated and purified for pharmaceutical peptide production (Figure 1) (Overton, 2014; Jozala et al., 2016). The purified proteins are screened for both chemical and biological properties for quality assurance. Chemical screening includes the testing of pH, solubility, percentage of active ingredients, whereas biological screening includes sterility test, endotoxin test, etc. Although biologics production from microorganisms is complicated, it is not as complicated as CBT production from living cells. Regardless, having fewer variables that need to be controlled, biologics production from microorganisms results in standardized and homogenous production of both target peptides and stock cells.

Difficulty in bioprocessing is increased when pharmaceutical proteins are produced using human, animal or plant cells. High-quality products in this case depend on the maintenance of high-quality cells and sterile conditions as these cells are generally less robust in culture, and tend to be more difficult to work with. In this case, cells transfected with the genes of interest are cultivated for a certain period of time after inoculation from a master cell bank (Lai et al., 2013; Tekoah et al., 2015). After expansion, cells are stored for future use or discarded after collecting the supernatant. The desired proteins or antibodies are separated, purified and concentrated. The isolated products are then checked for physical, chemical or biological properties similar to microbial peptide production to meet the regulatory agency’s criteria through quality assurance. A considerable benefit is that once the protein is produced, it again tends to be much more stable and characterizable compared to cells for CBT.

Stem cell or blood-derived product manufacturing is not as direct as the production of pharmaceutical proteins or vaccines (Figure 1). This is because cells are the product in CBT and they tend to be less robust in the face of perturbations and more vulnerable to changes in cell identity and gene expression. Manufacturing strategies for cell production can vary from source to source and can differ significantly based on autologous or allogeneic transplantation. The main general steps are the acquisition of tissue samples and cell isolation, initial cell purification, selection, activation and transduction, expansion of cells, differentiation, washing, harvesting and formulation, filling and cryopreservation, and finally storage and delivery to clinics (Roh et al., 2016). At every step, quality assurance and consideration for safety and efficacy are important for all CBT products manufactured for clinical application.

CBT products also differ from sources of tissue acquisition and target of diseases treated. For example, in CAR-T therapy, T-cells are isolated from patients’ blood, which contain abnormal levels of inhibitory factors and regulatory cells (Bellone et al., 1999; Gajewski et al., 2013) as patients are commonly also treated with chemo- and radiotherapies. Accordingly, heterogeneity can occur in the final products, which need much attention during the cell isolation step. Then initial cell culture is done for selection, activation or transduction of specific interest, in this case, the CAR gene. The transduced cells are then expanded in plate culture and stored in the master cell bank.

Cells are also screened for quality, safety and efficacy. Product potency is an important criterion to meet before releasing the product. For example, if a CBT product is applied for the CAR-T related cancer therapy, it needs to be examined for the secretion of cytotoxic cytokines (IFN-γ) and killing of target cells (Dudley et al., 2003). After passing all the steps of quality assurance, cells are stored or delivered to the clinic. Often cells need to be delivered in a timelier manner than other biologics and have a much less stable “shelf life”, which needs to be taken into serious consideration.

There is a further increase in the magnitude of process complexity, time, labor and cost when moving to CBT production from human hMSCs to hPSCs (Figure 1). MSCs are only slightly more complicated than T-cells. For biologics manufacturing from hMSCs, cells are expanded and stored in master cell banks after isolation and purification from patients’ bone marrow (Ullah et al., 2015). Then cells are genetically modified or expanded and differentiated into specific types of cells.

An interesting possibility being exploited with MSCs is that sometimes cell-derived bi-products can be used for clinical applications. For example, exosomes secreted from MSCs contain autocrine or paracrine signaling components (cytokines, RNAs, etc.) (Pisitkun et al., 2004; Valadi et al., 2007; NHLBI, 2009; Chen et al., 2010) and show immunological activities (Lai et al., 2011; Zhang et al., 2013; Lou et al., 2017). For this reason, it’s possible to consider value-added products, or creation of processes that can utilize such a potential secondary resource. MSC-derived exosomes are currently being studied for treating degenerative diseases (Chang et al., 2018; Yin et al., 2019). MSC-derived exosomes have recently been approved by US FDA for treating burn patients (PRWeb, 2018). For using exosomes in clinical applications, hMSCs are expanded and exosomes are purified by ultracentrifugation or size exclusion chromatography from culture medium. The benefit to this is that if the cell therapy itself is cGMP compliant, then it may be possible to have little added effort to extract a secondary cGMP compliant product in the form of exosomes. However, whether clinical applications use cells or cell-derived products, it needs go through a strict quality screening. Since here the product is cells, it needs to pass the *in vivo* biological tests for quality, efficacy and safety. After confirming the quality assurance, cells are cryopreserved or delivered to clinics.

hPSCs by contrast are very complicated; however, they have many benefits making theme an important cell choice for CBT. Being pluripotent, hPSCs have the ability to be differentiated into any cell type in the body. This provides advantages for therapies involving cells, other than T cells or MSCs that are not accessible via biopsy. Unlike T cells or MSCs, hPSCs do not senesce, making them very conducive to cell bio-banking (Zeng, 2007; Koch et al., 2013).

Biologics manufacturing from hESCs is impractical as isolation from human embryos has been unethical in many jurisdictions (Lo and Parham, 2009). Accordingly hESCs have been superseded by hiPSCs, which avoid such ethical barriers. Compared to hESCs which are subject to immune-rejection due to human leukocyte antigen (HLA) expression after differentiation (Taylor et al., 2005), iPSCs provide a better platform for autologous therapy because terminally-differentiated cells can be reprogrammed to desired cells using the four Yamanaka factors.

iPSCs also provide an alternative option of allogeneic treatment by creating haplotype biobank. HLA-typed biobank can help reduce both the rejection of grafted tissue, and the number of cell lines that are required to meet all populations in a given country (Zimmermann et al., 2012). Nakagawa et al. (2011) reported an integration-free iPSCs generation method that provides HLA-typed biobanking which match 20% of Japanese population.

In the case of biologics production from hiPSCs, the current paradigm is that cells are isolated from patients and reprogrammed into iPSCs using Yamanaka factors in conventional plate culture (Takahashi et al., 2007). As the Yamanaka factors contain the proto-oncogene, c-Myc, there are possibilities of increased genetic abnormalities from viral integration (Nakagawa et al., 2008, 2010). Currently, multiple methods of reprogramming of hiPSCs have been developed due to their unique limitations. Some methods that may be currently considered safer involve using mRNA, proteins, or cytokines although they have their own limitations as well, such as poor transduction efficiency (Kim et al., 2009; Warren et al., 2010). It is well established that these methods are less efficient than viral vectors for reprogramming. However, there are some efficient non-integrative viral vector approaches developed recently for reprogramming that we discuss later part of this study.

Depending on the final product and expected timelines, cell expansion is a very important consideration and area for considerable risk management analysis. After reprogramming, the cells are stored in the master cell bank or expanded for differentiation. Due to generally tighter time considerations in manufacture, application, and shelf-life, large-scale expansion is required in a sterile condition based on demand. This requires intensive consideration because it is a major rate-limiting step in the manufacturing of CBT products. The most important considerations for expansion on a large scale are: operational, economic, quality and safety (Table 2).

**TABLE 2:** Considerations for large-scale expansion of hPSCs in bioreactor culture.

Characteristics	What to consider
Operational considerations	• Culture system (2D/3D) • Manual or automatic operation • Process control and monitoring (online/offline) • Culture environment (temperature, pH, DO, pCO_2_ etc.) • Scalability (scale-up/scale-out) • Culture time • Culture vessel (single/multi-use) • Target final cell density (cells/mL) • Medium feeding regimen (once/twice in a day) • Prediction model
Economic considerations	• Medium • Resources (devices, labor, etc.) • Cell storage (cryopreservation) • Efficient cell lines • Indirect utilities
Considerations for quality and safety	• Quality control • cGMP compliant • Efficacy (*in vitro* and *in vivo*) • Harvest purity

Before large-scale expansion, it is important to consider operational design (2D or 3D) with manual or automatic operation (Jenkins and Farid, 2015). In order to obtain a large number of cells, bioreactors tend to be far superior to plate culture in total cell production (Table 1). Important parameters for operational consideration are online monitoring and control of process parameters (pH, DO, pCO2, etc.), as well as considering the shortest possible cultivation time. Due to the nature of 2D culture, it is often difficult to implement monitoring and control methods and therefore rely on operator know-how and standardization of process methods (such as changing media daily). However, by utilizing 3D vessels it is possible to automate or semi-automate monitoring and process parameters via onboard sensors, with the potential for savings in reagents and removing sources of error. For determining medium feeding regimes, a prediction model for medium consumption (glucose and glutamine) and production of toxic materials (lactic acid and ammonium) can be very useful (Galvanauskas et al., 2019). More data and higher quality data can lead to more effective decisions and use of advanced analytical tools. A single-use vessel is also a major operational consideration that increases expansion cost for cell-based products on a large scale. Although single-use vessel reduces contamination risk by eliminating cleaning procedures and its validation, the cost is not rather dominating here as the priority for the product safety is high. Since cellular products are costly, economic considerations are important for medium, efficient cell lines and other indirect utilities. Above all, product quality and safety are the most important consideration that will provide safe and efficient final product for CBT application.

After expansion, cells are harvested by separating them from the culture substratum of plate or microcarrier using enzymatic treatment or by changing temperature or pH (Yang et al., 2010; Guillaume-Gentil et al., 2011; Dou et al., 2012). For harvesting, aggregate culture in bioreactors may not necessarily require a detachment step (Bartosh et al., 2010; Amit et al., 2011; Larijani et al., 2011; Zweigerdt et al., 2011; Nath et al., 2018), however, microcarrier culture in bioreactor requires detachment step. Purified cells are formulated and checked for quality assurance. Quality assurance is carried out in three different stages: microbial contamination, chemical contamination and quality or potency. Microbial contamination is checked with different methods for bacteria, fungus or virus (Rayment and Williams, 2010; Goldring et al., 2011). A 14-day incubation of cell products for bacterial and fungal contamination is the most commonly used sterility test (Khuu et al., 2006; Hocquet et al., 2014). Chemical testing includes checking molecules that accompany the culture medium or other factors used during isolation, expansion and storage. The LAL test for bacterial endotoxin is a common chemical test. An automated 15-min test to determine endotoxin in CBT products has now been developed in accordance with FDA regulations (Gee et al., 2008). Other chemical testing concerns examine residual proteins of different origins, serum and other harmful cell processing particles.

Quality is the main concern in CBT products, especially when cell growth is a requirement. A cell viability test is therefore performed to determine live or dead cells in the product using a variety of staining methods. It is also useful to determine the biological activity of CBT products (Choi et al., 2011; Schellenberg et al., 2013). Pre-release product potency is an important criterion to meet. For example, the final products for hPSCs are differentiated cells, wherein the potency should be checked through transplantation into disease models.

Strict quality control is imperative for products derived from hPSC before transplantation to patients, as there is a high risk of transferring oncogenes to patients. In Japan, a clinical trial was halted in 2015 when treating AMD with autologous retinal pigmented epithelial cells derived from hiPSC due to genetic abnormality (Garber, 2015). Since genetic abnormalities occur in products derived from hiPSCs from reprogramming to finally differentiated cells (Rohani et al., 2014), cells should be screened strictly for epigenetic signatures, karyotype, telomerase activity, and mitochondrial remodeling, and functional assays including teratoma formation and *in vitro* differentiation (Feng et al., 2010; Kim et al., 2010; Yehezkel et al., 2011; Rohani et al., 2018). Some of the other proposed quality tests include whole- genome sequencing, single- cell genome sequencing, epigenetic analysis, and DNA integrity testing to maximize patient safety.

Cells must be delivered to clinics immediately or stored for future use after the product quality assurance has been passed. If the cells are vitrified, cells are usually shipped to clinics on dry ice (−78°C) or in liquid nitrogen dry shippers (−160°C). The most commonly used cell storage technique is cryopreservation in liquid nitrogen at −196°C, which is adapted from the conventional stem cells banking (Thirumala et al., 2009; Hunt, 2011). For the better recovery of cryopreserved cells, slow-freezing and rapid thawing is generally highly applicable (Kurata et al., 1994; Moon et al., 2008). Recently Celikkan et al. (2019) developed a new media for transporting multipotent stromal cells using Ringer’s lactate-based transport media supplemented with human serum albumin that supported more than 90% cell survival after 6 h of transportation. Developing more robust methods and media is also important for long distance cell delivery.

## Standardization of Biologics Manufacturing in Bioreactors

Manufacturing of pharmaceutical proteins or other biological products consists of several steps from raw materials to finished products that may significantly compromise the quality of the product. They also reduce productivity and are prone to human errors. Different pharmaceutical companies have attempted integrated pharmaceutical production to overcome these disadvantages. One of the major attempts to fully integrate the cell processing system is being made by the Novartis-MIT Center for Continuous Production of Pharmaceutical Products (Bisson, 2008; Schaber et al., 2011). Genzyme^TM^ is also attempting to continuously produce pharmaceutical recombinant protein in bioreactors, wherein cell culture is being integrated into a single flow for product isolation and purification (Warikoo, 2011). In order to reduce cumbersome production steps and significantly reduce costs, process integrity is necessary. One such integrated system developed by Johnson and Johnson has recently been approved by the FDA for large-scale production of HIV drugs (FIERCE Pharma, 2016), which have been shown to reduce time and costs by one third compared to conventional batch processing.

**FIGURE 2:**
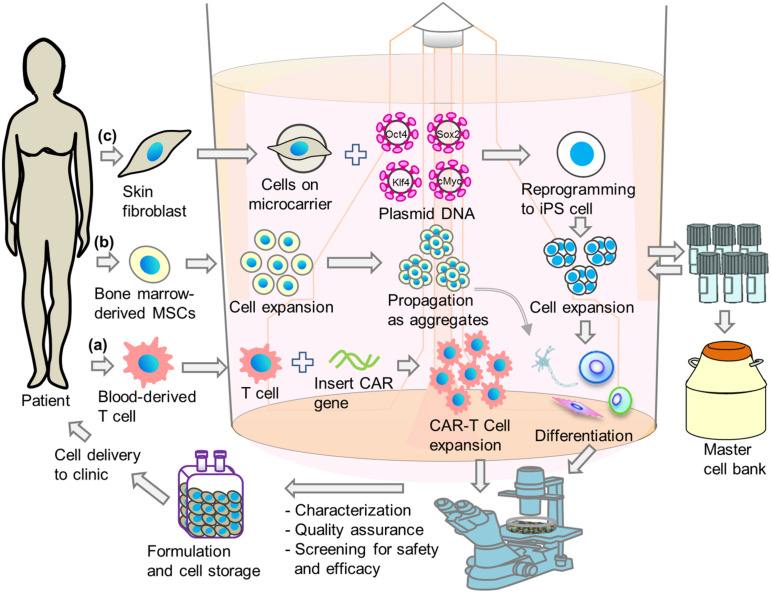
Schematic illustrations of integrated single-step cell manufacturing strategies in bioreactor culture for cell therapy applications. Cells are isolated from patient’s (a) blood or (b) bone-marrow, or (c) skin and genetically modified. After expansion, cells are stored in a master cell bank or differentiated directly in bioreactor. After performing characterization, quality assurance and screening for safety and efficacy, cells are delivered to hospital or stored in cell bank for future use.

The integration of production steps provides high product quality and safety and helps to overcome strict regulatory requirements by easing ability to obtain and retain cGMP compliance. In this context, the next sections will discuss how to integrate certain important basic steps in cell production, in particular genetic modification, cell reprogramming, expansion and differentiation in bioreactors to promote a single step approach for cell-based therapies utilizing T-cells, MSCs, and hPSCs (Figure 2).

### Standardization of Genetic Modifications in Bioreactors

Genetic modification has been applied for production of antibodies, proteins or other biotechnological drugs production for many years in the pharmaceutical industry. It has also been widely used in recent years to treat multiple incurable genetic diseases. For example, in a neurological disorder called adrenoleukodystrophy (ALD), there is a malfunction of oligodendrocytes and microglia, and genetic modification can directly affect disease outcome. A corrected gene is inserted into the patient-derived hPSCs and transplanted into the patient’s brain. The inserted hPSCs differentiate into microglia to promote myelin production in the patient’s brain that affects ALD outcomes (Cartier et al., 2009).

Sometimes patients are exposed indirectly to genetic modification. For example, in thalassemia, patient blood cells are extracted from the body are modified and enriched *ex vivo* in order to target specific antigens in the body of patients (Naldini, 2011). Other indirect genetic changes include modification of CAR or T cell receptors (TCR) in T- cells (Wang and Riviere, 2015), expression of CD40 ligand in dendritic cells (Kikuchi et al., 2000), etc. Genetically modified CAR-T cells can target antigens specifically and efficiently kill cancer cells (Song, 2013). CARs and TCRs are the most commonly used receptors for the activation of T cells (Kerkar, 2013). Many cell-based CAR-T therapies are now being developed for the treatment of advanced- stage lymphoma (Kochenderfer and Rosenberg, 2013), B-cell lymphoma (Kochenderfer et al., 2010) and other autoimmune diseases (Jethwa et al., 2014).

To transduce cells with desired genes, viral vectors are usually used as genetic cargos. Genetic transduction is a two-step process: preparing viral vectors and transducing cells to express the desired property. For their superior transduction efficiency, lentiviral and gamma-retroviral vectors are widely used. However, these vectors still currently have some issues, such as the ability to integrate into the host genome running the risk of mutation (Sakuma et al., 2012), which raises safety concerns (Yamashita et al., 2016).

Non-integrative viruses, such as, Sendai virus have proven to be useful for transient transfections, including cellular reprogramming. There are other non-viral approaches. DNA vectors can carry large cargo with less immunotoxicity and are easy to scale up (Hsu and Uludag, 2012). However, their use is less efficient than viral transduction. Non-viral cationic reagents have also been reported to successfully transfect cells with high efficiency (Hsu et al., 2018). Effective transduction using mRNA was also reported for reprogramming terminally differentiated cell to hiPSCs (Warren et al., 2010; Rohani et al., 2016). hiPSCs have also been reprogrammed using recombinant protein (Kim et al., 2009).

As viral vectors and/or DNA integration possess high risk of cancer, some methods for removing these vectors have been developed. One method is the piggyBac, a transposon system that was used to remove tandem Yamanaka factors from iPSCs after reprogramming (Woltjen et al., 2009). After CAR was incorporated into T-cells, the removal of transgenes used another transposon system called Sleeping Beauty, which successfully removed genetic scars from the transduced cells (Huls et al., 2013; Singh et al., 2013). Similarly, Cre excision of reprogramming genes via loxP sites has also resulted in transgene-free iPSCs (Sommer et al., 2010). Viral vectors deficient in integration are also good candidates for the production of transgene-free CBT by mutating viral integrase (Yáñez-Muñoz et al., 2006).

Genome engineering technologies are other choices for the addition, deletion or correction of genes in the CBT industry (Lombardo et al., 2007). Zinc-finger nucleases (ZFNs), clustered regulatory interspaced short palindromic repeats (CRISPR)/Cas endonucleases, or transcription-activator nucleases (TALENs) (Provasi et al., 2012) are the most commonly used targeting nucleases. CRISPR/Cas system has recently received much attention due to its broad use in the genome engineering of patient cells (Mullard, 2015). ZFNs are also popular for treating graft-versus-host disease in T-cell therapy (Gaj et al., 2013). In cellular reprogramming, a nuclease dead variant of Cas9 with a transcriptional *trans*-activator was recently used by activating the transcription factors Oct4 and Sox2, which maintained pluripotency and expressed the markers for the three germ layers (Liu et al., 2018).

Genetic modification is considered one of the rate-limiting steps in cell manufacturing industry. Current conventional methods make it more complicated because they are multi-step processes. Genetic modification in planar culture is also expensive, time-consuming and labor-intensive (Hsu et al., 2018). The bioreactor is a better platform for the production of large-scale genetically modified cells for commercial purposes (Figure 2). In adherent culture, reprogramming factors are generally transfected in order to generate iPSCs, and cells are then expanded in 2D or 3D, which is a two-step process. By integrating the genetic modification step into the bioreactor, a single-step process can be established that allows the production of cells in an automated and closed bioreactor system (Hsu et al., 2018).

In CAR-T CBT application, genetic modification is also a challenging step. After isolating cells from the blood sample of patients, selection and activation are done followed by expansion (Wang and Rivièrea, 2016). Finally, it is necessary to transduce cells with CAR or any other antigens depending on target diseases (Pampusch et al., 2020). Conventional genetic transduction methods are based on a planar culture wherein each step is carried out in an open culture system posing difficulties in maintaining cGMP.

A few steps have recently been integrated into the bag culture system, wherein selection, activation and expansion can be carried out in a single step using DynaMag^TM^ CTS^TM^ (Hollyman et al., 2009), while the Xuri^TM^ cell expansion system developed by GE Healthcare can expand cells in large numbers (Jin et al., 2012; Somerville et al., 2012). Although washing and concentrating the final product is integrated into the COBE^®^ 2991 system developed by Terumo BCT (Bajgain et al., 2014), the transduction step is not yet integrated into any of the above systems. The integration of the transduction step with the expansion and formulation will make CAR-T CBT straight forward and it is a good platform to carry out these steps in the bioreactor.

Miltenyi Biotech has developed a device called CliniMACS Prodigy^TM^ based on bag culture for CAR-T CBT. In an automated system (Terakura et al., 2012; Casati et al., 2013; Acker et al., 2016), this device integrated major steps in particular cell preparation, selection, activation, transduction, expansion, washing, and formulation. Such integration in the bioreactor will pave the way for the production of closed and automatic cell-based products. This device is also useful for cGMP-compliant production of dendritic cell for cell therapy applications (Erdmann et al., 2018). These systems are now beginning to prove their superiority in implementation of manufacturing protocols and cGMP compliance. Early data also suggests that adoption of such technologies even at an early stage may still be superior to conventional methods and offer returns on investments due to ability to adapt to multiple cell types and different processes (Terakura et al., 2012; Casati et al., 2013; Acker et al., 2016).

### Integration of Expansion and Differentiation in Bioreactors

Current manufacturing practices for cell-based products are multi-step: derivation, expansion, and differentiation. In this process, cells are isolated from any part of patient’s body. In case of MSCs, bone marrow-derived stromal cells are mostly used for clinical application. However, only a very limited number of MSCs can be isolated from specific sources, e.g., MSCs represent only approximately 0.01% of the total fraction of mononuclear cells in the bone marrow (Apel et al., 2013; Orozco et al., 2013). The clinical applications of hMSCs require 1–5 million cells per kilogram of body weight of patient (Meirelles and Nardi, 2003; Connick et al., 2012). In addition, it is important that the derived MSCs can differentiate properly. Therefore, bioprocess development for both expansion and differentiation is equally important.

Several clinical trials using MSCs are taking place worldwide and are increasing day by day. More than 200 clinical trials using MSCs were conducted in 2015 and 2016, which require intensified standardization of production practices (Carlsson et al., 2015). Typically, MSCs are expanded in plate culture and transplanted as whole cells or differentiated into lineage-specific cells for clinical application. However, plate culture is disadvantageous when the clinical trial requires large number of cells (Table 1). Bioreactor expansion of MSCs is required and can provide a large number of cells to exploit the remarkable potential of MSCs in therapeutic applications. When grown as three-dimensional aggregates or spheroids, MSCs show increased angiogenic, anti-inflammatory and immunomodulatory effects and improved stemness and survival after transplantation (Bunpetch et al., 2019). The dynamic culture of MSCs also increases the survivability, proliferation and paracrine effects.

Different configurations of dynamic bioreactors have been developed for MSC expansion according to specific cell types. Among them stirred suspension bioreactors (SSB), rotating wall vessel bioreactors (RWV), and perfusion bioreactors were widely used for the expansion of MSCs (Chen et al., 2006; Rodrigues et al., 2011; Hanley et al., 2014; Lechanteur et al., 2014; Lawson et al., 2016; Egger et al., 2018). To reduce heterogeneous shear stress, NASA has developed a RWV bioreactor (Somerville et al., 2012) which has been shown to be superior to SSB for MSC expansion and differentiation (Chen et al., 2006). Since MSCs have the capacity for multi-lineage differentiation, integration of expansion and differentiation could help to streamline manufacturing processes. Several researchers have reported the integration of expansion and differentiation in the derivation of osteogenic and adipogenic lineages in bioreactors (Chen et al., 2004; Dominici et al., 2006; Duijvestein et al., 2010; Hoch et al., 2012). MSCs have also been expanded and differentiated on microcarriers (see below) in stirred suspension bioreactors for deriving osteogenic lineages (Field et al., 1994; Mazzini et al., 2003; Goh et al., 2013; Shekaran et al., 2016).

As generation of hPSCs from terminally differentiated patient cells is complicated by several steps, the final products are at high risk of contamination. Therefore, as with any pharmaceutical grade medical product, it is also mandatory to maintain cGMP for CBT products (Sensebe et al., 2011; Heathman et al., 2015; Wuchter et al., 2015), which adds many additional complications in the cell production process. It is therefore necessary to standardize the system that can combine all these steps from derivation to final products. Bioreactors are a platform that has shown capability in filling this role (Figure 2).

The bioreactor platform is widely used for the large-scale expansion of hPSC-based CBT production because it is easy to operate and different physicochemical parameters can be regulated in a closed-system. Two groups have shown the bioreactor derivation of PSCs (Fluri et al., 2012; Shafa et al., 2012). Shafa et al. (2012) reported a significantly higher efficiency in bioreactor reprogramming compared to the planar culture. Since mesenchymal-epithelial transition (MET) is an important early step in cellular reprogramming (Samavarchi-Tehrani et al., 2010), transformed fibroblasts moved into the bioreactor form aggregates that are efficiently expanded in the bioreactor. Since fibroblasts are substrate dependent, bioreactor culture can promote the formation of aggregates and therefore cellular reprogramming.

After the bioreactor derivation of hPSCs, expansion and differentiation are the next major steps. A large number of cells are generally required for effective CBT, and can range anywhere from 10^8^ to 10^10^ cells per 70 kg patient (Serra et al., 2012). Obtaining such numbers of cells in plate culture is cumbersome because of growth surface limitation. The surface coating with extracellular matrix (ECM) poses a high risk in the production of clinical products because it is usually derived from animal sources. Recently, a discovery of recombinant ECM (laminin E8 fragment) has been developed that can be used efficiently in clinical applications (Miyazaki et al., 2012).

Automated planar culture systems have been established for the production of clinical-grade hPSCs. CompacT Selec^TM^ developed by TAP Biosystems is one of the notable automated systems for cell production. This system is based on T-flask design, which can accommodate ninety T175 flasks for cell expansion on a large scale. This robotic system can automatically perform all cell culture steps, cell counting, seeding, medium change, passage, plating and transient transfection. Such systems are not used for differentiation, however, because differentiation is a complicated process that needs several components to be added to the culture medium. This mainly disintegrates the process of expansion and differentiation in planar culture.

Except for microcarrier culture, cell expansion in bioreactors does not need surface coating. The bioreactor also provides sufficient availability of growth surface. In general, a single bioreactor (100 ml working volume) is sufficient to provide autologous CBT with a clinically relevant number of cells. For the expansion of hPSCs, several types of bioreactors are used (Wang et al., 2014) (Table 2).

Microcarriers must be coated with ECM for cell attachment in the bioreactor (Olmer et al., 2012; Lam et al., 2014; Fan et al., 2015; Badenes et al., 2016; Kropp et al., 2017) for anchorage-dependent expansion of hPSCs. Cells are harvested by removing them from the microcarrier using enzymatic treatment after large-scale expansion. Bioreactor expansion of hPSCs on microcarriers is problematic for clinical use, as separating the microcarrier from the final cell harvest requires an additional step. On the other hand, aggregate cultivation in bioreactors may not necessarily require a detachment step for harvesting (Bartosh et al., 2010; Amit et al., 2011; Larijani et al., 2011; Dou et al., 2012; Nath et al., 2018), and clinically relevant cell numbers has been produced as aggregate in a single bioreactor (Haraguchi et al., 2015; Rungarunlert et al., 2016; Nath et al., 2017, 2018) (Table 3).

**TABLE 3:** Summary of hPSCs expansion in stirred suspension bioreactor.

Cell types	Seeding density, (cells/mL, 10^5^)	Final density, (cells/mL, 10^5^)	Culture time (day)	Medium volume, (mL)	Bioreactor types	References
hESC	3.3	7.9	4	100	Bioreactor (DASGIP/Eppendorf)	Kempf et al., 2015
hESC	0.7	7	5	200	Gas permeable single use bag (Nipro)	Otsuji et al., 2014
hiPSC	3	10	6	100	Spinner flask (Cellspin, Integra Biosciences)	Haraguchi et al., 2015
hiPSC	4	16	5	50	Spinner flasks (Cellspin, Integra Bio-sciences)	Wang et al., 2013
hESC	2.5	20	6	60	Spinner flasks (50119114, Thermo Scientific)	Chen et al., 2011
hiPSC	4	15	7	100	Bioreactor (DASGIP/Eppendorf)	Olmer et al., 2012
hiPSC	3	12	7	100	Bioreactor (Cellspin, Integra Biosciences)	Abbasalizadeh et al., 2012
hESC	10	20	7	100	Spinner flask (IBS Integra Biosciences)	Zweigerdt et al., 2011
hESC	1	18	6	50	Spinner flasks (Integra Biosciences)	Amit et al., 2011
hESC	10	20	7	50	Spinner flask (Cellspin, IBS Integra Biosciences)	Singh et al., 2010
hESC	0.2	5	6	100	Bioreactor (NDS Technologies)	Krawetz et al., 2010
hESC	6	360	28	55	Slow-turning lateral vessel (Synthecon)	Gerecht-Nir et al., 2004
hiPSC	5	36	7	125	DASbox mini bioreactor system (Eppendorf AG, Hamburg, Germany)	Kropp et al., 2016
hiPSC	2	12	7	100	Spinner flask (Corning)	Kwok et al., 2018
hESC	6	35	5	100	Spinner flask, Bellco	Oh et al., 2009
hESC	2.5	3	10	125	Spinner flask (Croning)	Silva et al., 2015
hiPSC	5	5	5	100	Bioreactor (NDS Technologies)	Meng et al., 2017
hiPSC	1	45	8	100	Bioreactor (Able)	Nath et al., 2018

Size limitation is a major disadvantage in aggregate culture. As the aggregate size increases, the growth potential of the large aggregate decreases due to the limited diffusion of oxygen and nutrients (Nath et al., 2017). Maintenance of aggregate size is therefore an important point in order to maintain a high growth rate and high quality for CBT applications (Dou et al., 2012).

Bioreactor culture is a unique choice for CBT production because differentiation and expansion can be done in the same vessel. Bioreactors have been used to differentiate hPSCs into different cell types, especially for cardiac (Matsuura et al., 2012; Kempf et al., 2015; Rungarunlert et al., 2016), hepatic (Vosough et al., 2013; Park et al., 2014), and neural (Yan et al., 2016) lineages. In order to provide direct methods for clinical applications, it is important to integrate expansion and differentiation, and several reports have recently been published wherein expansion and differentiation have been integrated (Lam et al., 2014; Ting et al., 2014; Fonoudi et al., 2015; Haraguchi et al., 2015). The integration of derivation with expansion and differentiation, however, still faces complications and very few reports are available (Steiner et al., 2010).

Steiner et al. (2010) have reported integrating the derivation, propagation and differentiation of hESCs in the suspension culture where hESCs were isolated from the inner cell mass, and did not involve feeder cells or microcarriers. However, the integration of derivation, expansion and differentiation for personalized medicine, in particular for autologous or allogeneic CBT applications, has still not been achieved. This integration is necessary to overcome multi-step cell processing, which reduces the risk of contamination, saves cell processing time and reduces costs for the CBT manufacturing.

## Regulatory Constraints in CBT Manufacturing

With the advancement of cell processing facilities, several clinical trials have taken place in recent years using hPSCs (Lebkowski, 2011; Menasché et al., 2015; Schwartz et al., 2015; Trounson and McDonald, 2015; Mandai et al., 2017) which is triggering much attention from regulatory bodies on cGMP implementation in CBT manufacturing (Giancola et al., 2012). Since cell culture media contains various xenogens including source-derived virus and biochemicals (Martin et al., 2005; Cobo et al., 2008), CBT products need to be cultured in the xeno-free condition so that they are free from xeno-mediated infection or immune rejection according to regulatory agencies (Cimino et al., 2017). Currently both hESC and hiPSC lines have been derived following cGMP and deposited into national cell bank from various nations including China, Japan, United States, and United Kingdom, and their efficacy and safety has been tested using standard protocol (Hawkes, 2011; Ilic et al., 2012; Baghbaderani et al., 2015; Wang et al., 2015; Azuma and Yamanaka, 2016; Catapult, 2017). However, their differentiation capability into diverse cell lineages is yet to be tested although a few cell lines have already been tested in cGMP facilities (Kajiwara et al., 2012; Löhle et al., 2012; Heslop et al., 2017; Rao et al., 2018; Blackford et al., 2019; Shafa et al., 2019). Therefore, facilitating cGMP-compliant manufacturing and establishing standard operating procedures (SOP) according to regulatory agencies is a prime concern for CBT products.

cGMP is a regulatory framework overseen at the governmental level meant to establish basic minimum standards in safety, efficacy, and standardization of many types of products. All CBTs must meet regulatory requirements to be approved for sale and marketing. This is the end goal of clinical trials, to make a therapy available for wide scale implementation, and entry into worldwide markets. One of the first considerations is to begin engaging with the national regulatory board as soon as there even a thought about potential commercialization.

Approval from two of the most stringent regulatory organizations: USFDA, and the European Medicines Agency (EMA) that allows for easy entry into virtually every other market in the world (Rehakova et al., 2020). The intended application of human cells, tissues, and cellular and tissue-based products (HCT/P) are regulated by FDA’s Center for Biologics Evaluation and Research (CBER). However, FDA is intrinsically concerned about stem cell-related therapeutic products implementation, transplantation and infusion into patient since the cells may change their cellular properties after being expanded outside of body (Reisman and Adams, 2014). The EMA is generally considered the most stringent and well defined regulatory board for CBTs, and usually, if these standards are met, it will lead to the least problems in transferring to other markets, barring other legal issues such as patents and intellectual property rights.

Without engagement with regulatory boards, the CBT products have less chance of avoiding common pitfalls that have led to efficacious products not making it to market, even with good supporting data. This is important because, as mentioned, regulatory standards can differ across national lines. As the field is relatively new and advancing at a fast pace, regulators have been left to working with companies on a case-by-case basis to establish guidelines, and best practices that are in line with the stringency of cGMP standards in other areas (Bedford et al., 2018). This has led to the publishing of various guidance documents rather than overly strict ordinances, which acknowledge the inherent variability in these products, both between types (protein, whole cell, gene, etc.), and even between lots of the same product (European Medicines Agency, 2020; U.S. Food and Drug Administration, 2020).

It is well understood that CBTs inherently carry a higher level of heterogeneity, and difficult to control factors than chemical reactions that can produce extremely homogenous batches of classic pharmaceuticals. As part of this, regulators such as, Health Canada are often eager to work with potential CBT companies and academic institutions to develop guidelines based on the best current science available, and the unique product under review, while also helping to navigate the difficult regulatory terrain to higher phase studies and commercialization.

The second most important consideration is to hire or assign a team member to focus mainly on the navigation of regulation immediately. It is advisable that this be their main task, as the beginning stages are the most important. In CBTs, it is said that “the process is the product” and if the project is taken too far without this consideration it can be virtually impossible or at least economically unlikely to be able to recover after having to go back to earlier stages because details were overlooked. The unavoidable nature of GMP in CBTs is that even sometimes a seemingly simple laboratory procedure must become a multi-page SOP document covering literally every detail of the procedure. These documents then become the trail of documentation, and the very process itself, which is to be adhered to in manufacturing and available for regulatory review.

It is very important to attempt to begin to think about cGMP-like standards as soon as even the idea of a potential commercialization begins to materialize. Often this can be as early as pre-clinical, depending on the data, though perhaps more reasonably around phase 1 trials. For example, Health Canada requires increasing GMP compliance, and increasingly stringent manufacturing controls as trials advance although a manufacturing establishment license is not required while the product is still under any phase of clinical trials (Government of Canada, 2015). The FDA also requires GMP compliance in Investigational New Drug (IND) assessments at phase 1, and stringency increases in manufacturing establishment licensing from phase 2 onwards (US FDA, 2008). Finally, the EMA requires manufacturing authorization and compliance with established GMP regulations for all stages of clinical trial development, with “inspections performed by a competent authority/qualified person (QP) of a member state” (Bedford et al., 2018).

As one can see, in the United States and Canada an early phase can be “boutique” in having each treatment individually created under standard laboratory proper conditions and very carefully controlled, whereas this is not necessarily the case in Europe. Therefore, with increasing enrolment as trials continue a GMP-like approach should be adopted in every economically and physically feasible manner throughout the process as required. This includes making changes such as using “clinical grade” versus “laboratory grade” reagents, designing a robust manufacturing pipeline, considering quality and assurance guidelines, and creating in-depth documentation to regulatory standards to name merely a few requirements. It should be considered that any change in reagent, manufacturer of said reagents, processes, etc. carries a large weight as there is absolutely a chance of a change in associated outcomes and requirements.

One of the reasons bioreactors and automated processes are extremely useful in meeting GMP standards in CBTs is that, informally, some key aspects can be summarized as: the less manipulations in the process, and the fewer hands that touch to product, the better. Let us recall the phrase “the process is the product,” this is essential to remember for CBTs. If the process is not correctly implemented in a GMP-like fashion, it does not matter if the product works as intended. It will be required to go back the drawing board to make the bio-process compliant. This increases the time and cost even further.

It may be the case that for the near-future CBTs will only be successful in smaller-scale manufacturing, and smaller lots due to the challenges with characterization and standardization associated with whole cell populations. This may require smaller, more flexible operations spread across nations rather than mega-manufacturing centers as is currently the case with standard pharmaceuticals. It is prudent to consider de-centralized models of production in order to achieve profitability and market penetration.

CBTs often require more flexibility in production as there are finer windows of effectiveness and therapeutic application, as well as, inherently less ability to control and properly assess safety and efficacy in increasing scale. This may lead to needing to find ways to adapt similar processes across multiple pipelines in order to flexibly meet supply and demand based on various ailments and treatments. The upside of this is that integrated automated processes and bioreactor technologies tend to be far easier to scale up and down as required. Further advances in computing technology, basic biomarker discovery, cell characterization, and onboard or automated monitoring equipment will be essential in decreasing costs and increasing capacity, allowing for CBTs to become more and more ubiquitous in improving human health.

## Concluding Remarks and Future Directions

Application of CBT increases day by day and several clinical studies are continuing to treat incurable diseases. Manufacturing facilities should be compatible with the growing need for cell-based products to meet market demand by providing safe and effective cell-based products. Since current production systems have several disadvantages particularly multi-stage processing, which poses a high risk of contamination, long processing times, and increased production costs, a more straightforward cGMP-compliant system is needed. Bioreactor-based cell production systems can provide cell-based products with single step, easing the practice of cGMP for CBT production. The integration of various steps in bioreactors: derivation, genetic modification, expansion, and differentiation, will pave the way for the future of CBT manufacturing. The integrated production of cell biologics in bioreactor will significantly reduce the risk of contamination, and cell-processing time providing a cost-effective platform for CBTs.

## Author Contributions

SN and DR perceived the concept and design. SN and LH wrote the manuscript. DR revised and approved the manuscript. All authors contributed to the article and approved the submitted version.

## Conflict of Interest

The authors declare that the research was conducted in the absence of any commercial or financial relationships that could be construed as a potential conflict of interest.

## References

[B1] Abbasalizadeh S. Larijani M. R. Samadian A. Baharvand H. (2012). Bioprocess development for mass production of size-controlled human pluripotent stem cell aggregates in stirred suspension bioreactor. *Tissue Eng. Part C Methods* 18 831–851. 10.1089/ten.tec.2012.0161 22559864

[B2] Acker J. P. Marks D. C. Sheffield W. P. (2016). Quality assessment of established and emerging blood components for transfusion. *J. Blood Transfus.* 2016:4860284. 10.1155/2016/4860284 28070448 PMC5192317

[B3] Amit M. Laevsky I. Miropolsky Y. Shariki K. Peri M. Itskovitz-Eldor J. (2011). Dynamic suspension culture for scalable expansion of undifferentiated human pluripotent stem cells. *Nat. Protoc.* 6 572–579. 10.1038/nprot.2011.325 21527915

[B4] Apel M. Brüning M. Granzin M. Essl M. Stuth J. Blaschke J. (2013). Integrated clinical scale manufacturing system for cellular products derived by magnetic cell separation, centrifugation and cell culture. *Chem. Ing. Tech.* 85 103–110. 10.1002/cite.201200175

[B5] Attwood S. W. Edel M. J. (2019). iPS-Cell technology and the problem of genetic instability-can It ever be safe for clinical use? *J. Clin. Med.* 8:288. 10.3390/jcm8030288 30823421 PMC6462964

[B6] Azuma K. Yamanaka S. (2016). Recent policies that support clinical application of induced pluripotent stem cell-based regenerative therapies. *Regen. Ther.* 4 36–47. 10.1016/j.reth.2016.01.009 31245486 PMC6581825

[B7] Badenes S. M. Fernandes T. G. Rodrigues C. A. V. Diogo M. M. Cabral J. M. S. (2016). Microcarrier-based platforms for in vitro expansion and differentiation of human pluripotent stem cells in bioreactor culture systems. *J. Biotechnol.* 234 71–82. 10.1016/j.jbiotec.2016.07.02 27480342

[B8] Baghbaderani B. A. Tian X. Neo B. H. Burkall A. Dimezzo T. Sierra G. (2015). cGMP-Manufactured human induced pluripotent stem cells are available for pre-clinical and clinical applications. *Stem Cell Rep.* 5 647–659. 10.1016/j.stemcr.2015.08.015 26411904 PMC4624993

[B9] Bajgain P. Mucharla R. Wilson J. Welch D. Anurathapan U. Liang B. (2014). Optimizing the production of suspension cells using the G-Rex ‘M’ series. *Mol. Ther. Methods Clin. Dev.* 1:14015. 10.1038/mtm.2014.15 26015959 PMC4362380

[B10] Bartosh T. J. Ylöstalo J. H. Mohammadipoor A. Bazhanov N. Coble K. Claypool K. (2010). Aggregation of human mesenchymal stromal cells (MSCs) into 3D spheroids enhances their antiinflammatory properties. *Proc. Natl. Acad. Sci. U.S.A.* 107 13724–13729. 10.1073/pnas.1008117107 20643923 PMC2922230

[B11] Baum C. (2007). Insertional mutagenesis in gene therapy and stem cell biology. *Curr. Opin. Hematol.* 14 337–342. 10.1097/MOH.0b013e3281900f01 17534158

[B12] Bedford P. Jy J. Collins L. Keizer S. (2018). Considering Cell Therapy Product “Good Manufacturing Practice” Status. *Front. Med.* 5:118. 10.3389/fmed.2018.00118 29761103 PMC5936751

[B13] Bellone G. Turletti A. Artusio E. Mareschi K. Carbone A. Tibaudi D. (1999). Tumor-associated transforming growth factor-beta and interleukin-10 contribute to a systemic Th2 immune phenotype in pancreatic carcinoma patients. *Am. J. Pathol.* 155 537–547. 10.1016/S0002-9440(10)65149-8 10433946 PMC1866873

[B14] Bennett C. (2018). Available online at: https://www.genengnews.com/insights/cell-therapy-manufacturing-the-supply-chain-challenge/ (accessed April, 2020).

[B15] Bisson W. (2008). “Continuous manufacturing – the ultra-lean way of manufacturing,” in *Proceedings of the ISPE Innovations in Process Technology for Manufacture of APIs and BPCs*, Copenhagen.

[B16] Blackford S. Ng S. S. Segal J. M. King A. Austin A. L. Kent D. (2019). Validation of current good manufacturing practice compliant human pluripotent stem cell-derived hepatocytes for cell-based therapy. *Stem Cells Transl. Med.* 8 124–137. 10.1002/sctm.18-0084 30456803 PMC6344902

[B17] Bunpetch V. Zhang Z.-Y. Zhang X. Han S. Zongyou P. Wu H. (2019). Strategies for MSC expansion and MSC-based microtissue for bone regeneration. *Biomaterials* 196 67–79. 10.1016/j.biomaterials.2017.11.023 29602560

[B18] Carlsson P. O. Schwarcz E. Korsgren O. Le Blanc K. (2015). Preserved β-cell function in type 1 diabetes by mesenchymal stromal cells. *Diabetes* 64 587–592. 10.2337/db14-0656 25204974

[B19] Cartier N. Hacein-Bey-Abina S. Bartholomae C. C. Veres G. Schmidt M. Kutschera I. (2009). Hematopoietic stem cell gene therapy with a lentiviral vector in Xlinked adrenoleukodystrophy. *Science* 326 818–823. 10.1126/science.1171242 19892975

[B20] Casati A. Varghaei-Nahvi A. Feldman S. A. Assenmacher M. Rosenberg S. A. Dudley M. E. (2013). Clinical-scale selection and viral transduction of human naive and central memory CD8+ T cells for adoptive cell therapy of cancer patients. *Cancer Immunol. Immunother.* 62 1563–1573. 10.1007/s00262-013-1459-x 23903715 PMC6348480

[B21] Catapult C. G. T. (2017). *Early Seed Lot and Clinical Grade iPS Cell Line from the Cell and Gene Therapy Catapult.* Available at https://ct.catapult.org.uk/clinical-grade-iPS-cell-line (accessed April 5, 2018).

[B22] Celikkan F. T. Mungan C. Sucu M. Ulus A. T. Cinar O. Ili E. G. (2019). Optimizing the transport and storage conditions of current Good Manufacturing Practice -grade human umbilical cord mesenchymal stromal cells for transplantation (HUC-HEART Trial). *Cytotherapy* 21 64–75. 10.1016/j.jcyt.2018.10.010 30455106

[B23] Chang Y. H. Wu K. C. Harn H. J. Lin S. Z. Ding D. C. (2018). Exosomes and stem cells in degenerative disease diagnosis and therapy. *Cell Transplant.* 27 349–363. 10.1177/0963689717723636 29692195 PMC6038041

[B24] Chen G. Gulbranson D. R. Hou Z. Bolin J. M. Ruotti V. Probasco M. D. (2011). Chemically defined conditions for human iPSC derivation and culture. *Nat. Methods* 8 424–429. 10.1038/nmeth.1593 21478862 PMC3084903

[B25] Chen S. L. Fang W. W. Ye F. Liu Y. H. Qian J. Shan S. J. (2004). Effect on left ventricular function of intracoronary transplantation of autologous bone marrow mesenchymal stem cell in patients with acute myocardial infarction. *Am. J. Cardiol.* 94 92–95. 10.1016/j.amjcard.2004.03.034 15219514

[B26] Chen T. S. Lai R. C. Lee M. M. Choo A. B. Lee C. N. Lim S. K. (2010). Mesenchymal stem cell secretes microparticles enriched in pre-microRNAs. *Nucleic Acids Res.* 38 215–224. 10.1093/nar/gkp857 19850715 PMC2800221

[B27] Chen X. Xu H. Wan C. McCaigue M. Li G. (2006). Bioreactor expansion of human adult bone marrow-derived mesenchymal stem cells. *Stem Cells* 24 2052–2059. 10.1634/stemcells.2005-0591 16728560

[B28] Choi W. H. Choi B. H. Min B. H. Park S. R. (2011). Low-intensity ultrasound increased colony forming unit-fibroblasts of mesenchymal stem cells during primary culture. *Tissue Eng. Part C Methods* 17 517–526. 10.1089/ten.TEC.2010.0231 21171932

[B29] Cimino M. Gonçalves R. M. Barrias C. C. Martins M. C. L. (2017). Xeno-free strategies for safe human mesenchymal stem/stromal cell expansion: supplements and coatings. *Stem Cells Int.* 2017:6597815. 10.1155/2017/6597815 29158740 PMC5660800

[B30] ClinicalTrials.gov (2020). Available online at: https://clinicaltrials.gov (accessed October 8, 2020).

[B31] Cobo F. Navarro J. M. Herrera M. I. Vivo A. Porcel D. Hernández C. (2008). Electron microscopy reveals the presence of viruses in mouse embryonic fibroblasts but neither in human embryonic fibroblasts nor in human mesenchymal cells used for hESC maintenance: toward an implementation of microbiological quality assurance program in stem cell banks. *Cloning Stem Cells* 10 65–74. 10.1089/clo.2007.0020 18241120

[B32] Connick P. Kolappan M. Crawley C. Webber D. J. Patani R. Michell A. W. (2012). Autologous mesenchymal stem cells for the treatment of secondary progressive multiple sclerosis: an open-label phase 2a proof-of-concept study. *Lancet Neurol.* 11 150–156. 10.1016/S1474-4422(11)70305-2 22236384 PMC3279697

[B33] Cyranoski D. (2018). ‘Reprogrammed’ stem cells implanted into patient with Parkinson’s disease. *Nature* 563 1–2. 10.1038/d41586-018-07407-9

[B34] Davie N. L. Brindley D. A. Culme-Seymour E. J. Mason C. (2012). Streaming cell therapy manufacture. *Bioprocess Int.* 10 24–29.

[B35] Dominici M. Le Blanc K. Mueller I. Slaper-Cortenbach I. Marini F. C. Krause D. S. (2006). Minimal criteria for defining multipotent mesenchymal stromal cells. The International Society for Cellular Therapy position statement. *Cytotherapy* 8 315–317. 10.1080/14653240600855905 16923606

[B36] Dou X. Q. Yang X. M. Li P. Zhang Z. G. Schönherr H. Zhanga D. (2012). Novel pH responsive hydrogels for controlled cell adhesion and triggered surface detachment. *Soft Matter* 8 9539–9544. 10.1039/C2SM26442K

[B37] Dudley M. E. Wunderlich J. R. Shelton T. E. Even J. Rosenberg S. A. (2003). Generation of tumor-infiltrating lymphocyte cultures for use in adoptive transfer therapy for melanoma patients. *J. Immunother.* 26 332–342. 10.1097/00002371-200307000-00005 12843795 PMC2305721

[B38] Duijvestein M. Vos A. C. Roelofs H. Wildenberg M. E. Wendrich B. B. Verspaget H. W. (2010). Autologous bone marrow-derived mesenchymal stromal cell treatment for refractory luminal Crohn’s disease: results of a phase I study. *Gut* 59 1662–1669. 10.1136/gut.2010.215152 20921206

[B39] Egger D. Tripisciano C. Weber V. Dominici M. Kasper C. (2018). Dynamic cultivation of mesenchymal stem cell aggregates. *Bioengineering* 5:48. 10.3390/bioengineering5020048 29921755 PMC6026937

[B40] Erdmann M. Uslu U. Wiesinger M. Brüning M. Altmann T. Strasser E. (2018). Automated closed-system manufacturing of human monocyte-derived dendritic cells for cancer immunotherapy. *J. Immunol. Methods* 463 89–96. 10.1016/j.jim.2018.09.012 30266448

[B41] European Medicines Agency (2020). *Multidisciplinary: Cell Therapy and Tissue Engineering.* Available online at: http://www.ema.europa.eu/ema/index.jsp?curl=pages/regulation/general/general_content_000405.jsp&mid=WC0b01ac058002958a (accessed April 5, 2020).

[B42] Fan Y. Wu J. Ashok P. Hsiung M. Tzanakakis E. S. (2015). Production of human pluripotent stem cell therapeutics under defined xeno-free conditions: progress and challenges. *Stem Cell Rev.* 11 96–109. 10.1007/s12015-014-9544-x 25077810 PMC4312540

[B43] Feng Q. Lu S. J. Klimanskaya I. Klimanskaya I. Gomes I. Kim D. (2010). Hemangioblastic derivatives from human induced pluripotent stem cells exhibit limited expansion and early senescence. *Stem Cells* 28 704–712. 10.1002/stem.321 20155819

[B44] Field R. E. Buchanan J. A. Copplemans M. G. Aichroth P. M. (1994). Bone-marrow transplantation in Hurler’s syndrome. Effect on skeletal development. *J. Bone Joint Surg. Br.* 76 975–981. 7983131

[B45] FIERCE Pharma (2016). *FDA Urges Companies to Get on Board with Continuous Manufacturing.* Available online at: http://www.fiercepharma.com/manufacturing/fda-urges-companies-to-get-on-board-continuous-manufacturing (accessed April 14, 2016).

[B46] Fluri D. A. Tonge P. D. Song H. Baptista R. P. Shakiba N. Shukla S. (2012). Derivation, expansion and differentiation of induced pluripotent stem cells in continuous suspension cultures. *Nat. Methods* 9 509–516. 10.1038/nmeth.1939 22447133 PMC4954777

[B47] Fonoudi H. Ansari H. Abbasalizadeh S. Larijani M. R. Kiani S. Hashemizadeh S. (2015). A universal and robust integrated platform for the scalable production of human cardiomyocytes from pluripotent stem cells. *Stem Cells Transl. Med.* 4 1482–1494. 10.5966/sctm.2014-0275 26511653 PMC4675501

[B48] Gaj T. Gersbach C. A. Barbas C. F. (2013). ZFN, TALEN, and CRISPR/Cas-based methods for genome engineering. *Trends Biotechnol.* 31 397–405. 10.1016/j.tibtech.2013.04.004 23664777 PMC3694601

[B49] Gajewski T. F. Schreiber H. Fu Y. X. (2013). Innate and adaptive immune cells in the tumor microenvironment. *Nat. Immunol.* 14 1014–1022. 10.1038/ni.2703 24048123 PMC4118725

[B50] Galat V. Galat Y. Perepitchka M. Jennings L. J. Iannaccone P. M. Hendrix M. J. (2016). Transgene reactivation in induced pluripotent stem cell derivatives and reversion to pluripotency of induced pluripotent stem cell-derived mesenchymal stem cells. *Stem Cells Dev.* 25 1060–1072. 10.1089/scd.2015.0366 27193052 PMC4939377

[B51] Galvanauskas V. Simutis R. Nath S. C. Kino-Oka M. (2019). Kinetic modeling of human induced pluripotent stem cell expansion in suspension culture. *Regen. Ther.* 12 88–93. 10.1016/j.reth.2019.04.007 31890771 PMC6933447

[B52] Garber K. (2015). RIKEN suspends first clinical trial involving induced pluripotent stem cells. *Nat. Biotechnol.* 33 890–891. 10.1038/nbt0915-890 26348942

[B53] Gee A. P. Sumstad D. Stanson J. Watson P. Proctor J. Kadidlo D. (2008). A multicenter comparison study between the Endosafe PTS rapid-release testing system and traditional methods for detecting endotoxin in cell-therapy products. *Cytotherapy* 10 427–435. 10.1080/14653240802075476 18574775 PMC2518960

[B54] Gerecht-Nir S. Cohen S. Itskovitz-Eldor J. (2004). Bioreactor cultivation enhances the efficiency of human embryoid body (hEB) formation and differentiation. *Biotechnol. Bioeng.* 86 493–502. 10.1002/bit.20045 15129432

[B55] Giancola R. Bonfini T. Iacone A. (2012). Cell therapy: cGMP facilities and manufacturing. *Muscles Ligaments Tendons J.* 2 243–247. 23738304 PMC3666518

[B56] Goh T. K. Zhang Z. Y. Chen A. K. Reuveny S. Choolani M. Chan J. K. (2013). Microcarrier culture for efficient expansion and osteogenic differentiation of human fetal mesenchymal stem cells. *Biores. Open Access* 2 84–97. 10.1089/biores.2013.0001 23593561 PMC3620494

[B57] Goldring C. E. Duffy P. A. Benvenisty N. Andrews P. W. Ben-David U. Eakins R. (2011). Assessing the safety of stem cell therapeutics. *Cell Stem Cell* 8 618–628. 10.1016/j.stem.2011.05.012 21624806

[B58] Gore A. Li Z. Fung H. L. Young J. E. Agarwal S. Antosiewicz-Bourget J. (2011). Somatic coding mutations in human induced pluripotent stem cells. *Nature* 471 63–67. 21368825 10.1038/nature09805 PMC3074107

[B59] Government of Canada (2015). *Health Canada Guidance Document: Preparation of Clinical Trial Applications for use of Cell Therapy Products in Humans.* Available online at: https://www.canada.ca/en/health-canada/services/drugs-health-products/drug-products/applications-submissions/guidance-documents/clinical-trials/guidance-document-preparation-clinical-trial-applications-use-cell-therapy-products-humans.html (accessed April 6, 2020).

[B60] Guhr A. Kobold S. Seltmann S. Seiler Wulczyn A. E. M. Kurtz A. Löser P. (2018). Recent trends in research with human pluripotent stem cells: impact of research and use of cell lines in experimental research and clinical trials. *Stem Cell Rep.* 11 485–496. 10.1016/j.stemcr.2018.06.012 30033087 PMC6092712

[B61] Guillaume-Gentil O. Semenov O. V. Zisch A. H. Zimmermann R. Voros J. Ehrbar M. (2011). pH-controlled recovery of placenta-derived mesenchymal stem cell sheets. *Biomaterials* 3 4376–4384. 10.1016/j.biomaterials.2011.02.058 21458856

[B62] Hanley P. J. Mei Z. Durett A. G. Cabreira-Harrison M. Klis M. Li W. (2014). Efficient manufacturing of therapeutic mesenchymal stromal cells with the use of the Quantum Cell Expansion System. *Cytotherapy* 16 1048–1058. 10.1016/j.jcyt.2014.01.417 24726657 PMC4087082

[B63] Haraguchi Y. Matsuura K. Shimizu T. Yamato M. Okano T. (2015). Simple suspension culture system of human iPS cells maintaining their pluripotency for cardiac cell sheet engineering. *J. Tissue Eng. Regen. Med.* 9 1363–1375. 10.1002/term.1761 23728860

[B64] Hawkes N. (2011). Clinical grade stem cells are created by scientists in London. *BMJ* 343:d8001. 10.1136/bmj.d8001 22162294

[B65] Heathman T. R. J. Nienow A. W. McCall M. J. Coopman K. Kara B. Hewitt C. J. (2015). The translation of cell-based therapies: clinical landscape and manufacturing challenges. *Regen. Med.* 10 49–64. 10.2217/rme.14.73 25562352

[B66] Heslop J. A. Kia R. Pridgeon C. S. Sison-Young R. L. Liloglou T. Elmasry M. (2017). Donor-Dependent and Other Nondefined Factors have Greater Influence on the Hepatic Phenotype than the Starting Cell Type in Induced Pluripotent Stem Cell Derived Hepatocyte-Like Cells. *Stem Cells Transl. Med.* 6 1321–1331. 10.1002/sctm.16-0029 28456008 PMC5442714

[B67] Hoch A. I. Binder B. Y. Genetos D. C. Leach J. K. (2012). Differentiation-dependent secretion of proangiogenic factors by mesenchymal stem cells. *PLoS One* 7:e35579. 10.1371/journal.pone.0035579 22536411 PMC3334972

[B68] Hocquet D. Sauget M. Roussel S. Malugani C. Pouthier F. Morel P. (2014). Validation of an automated blood culture system for sterility testing of cell therapy products. *Cytotherapy* 16 692–698. 10.1016/j.jcyt.2013.09.005 24210785

[B69] Hollyman D. Stefanski J. Przybylowski M. Bartido S. Borquez-Ojeda O. Taylor C. (2009). Manufacturing validation of biologically functional T cells targeted to CD19 antigen for autologous adoptive cell therapy. *J. Immunother.* 32 169–180. 10.1097/CJI.0b013e318194a6e8 19238016 PMC2683970

[B70] Hsu C. Y. M. Uludag H. (2012). Nucleic-acid based gene therapeutics: delivery challenges and modular design of non-viral gene carriers and expression cassettes to overcome intracellular barriers for sustained targeted expression. *J. Drug Target* 20 301–328. 10.3109/1061186X.2012.655247 22303844

[B71] Hsu C. Y. M. Walsh T. Borys B. Kallos M. Rancourt D. E. (2018). An integrated approach towards the bio-manufacturing of engineered cell therapy products in a continuous stirred suspension bioreactor. *Mol. Ther. Methods Clin. Dev.* 9 376–389. 10.1016/j.omtm.2018.04.007 30038941 PMC6054699

[B72] Huls M. H. Figliola M. J. Dawson M. J. Olivares S. Kebriaei P. Shpall E. J. (2013). Clinical application of Sleeping Beauty and artificial antigen presenting cells to genetically modify T cells from peripheral and umbilical cord blood. *J. Vis. Exp.* 72:e50070. 10.3791/50070 23407473 PMC3596954

[B73] Hunt C. J. (2011). Cryopreservation of human stem cells for clinical application: a review. *Transfus. Med. Hemother.* 38 107–123. 10.1159/000326623 21566712 PMC3088734

[B74] Ilic D. Stephenson E. Wood V. Jacquet L. Stevenson D. Petrova A. (2012). Derivation and feeder-free propagation of human embryonic stem cells under xeno-free conditions. *Cytotherapy* 14 122–128. 10.3109/14653249.2011.623692 22029654

[B75] Jenkins M. J. Farid S. S. (2015). Human pluripotent stem cell-derived products: advances towards robust, scalable and cost-effective manufacturing strategies. *Biotechnol. J.* 10 83–95. 10.1002/biot.201400348 25524780 PMC4674985

[B76] Jethwa H. Adami A. A. Maher J. (2014). Use of gene-modified regulatory T-cells to control autoimmune and alloimmune pathology: Is now the right time? *Clin. Immunol.* 150 51–63. 10.1016/j.clim.2013.11.004 24333533

[B77] Jin J. Sabatino M. Somerville R. Wilson J. R. Dudley M. E. Stroncek D. F. (2012). Simplified method of the growth of human tumor infiltrating lymphocytes in gas permeable flasks to numbers needed for patient treatment. *J. Immunother.* 35 283–292. 10.1097/CJI.0b013e31824e801f 22421946 PMC3315105

[B78] Jozala A. F. Geraldes D. C. Tundisi L. L. Feitosa V. A. Breyer C. A. Cardoso S. L. (2016). Biopharmaceuticals from microorganisms: from production to purification. *Braz. J. Microbiol.* 47 51–63. 10.1016/j.bjm.2016.10.007 27838289 PMC5156500

[B79] Kajiwara M. Aoi T. Okita K. Takahashi R. Inoue H. Takayama N. (2012). Donor-dependent variations in hepatic differentiation from human-induced pluripotent stem cells. *Proc. Natl. Acad. Sci. U.S.A.* 109 12538–12543. 10.1073/pnas.1209979109 22802639 PMC3411998

[B80] Kempf H. Kropp C. Olmer R. Martin U. Zweigerdt R. (2015). Cardiac differentiation of human pluripotent stem cells in scalable suspension culture. *Nat. Protoc.* 10 1345–1361. 10.1038/nprot.2015.089 26270394

[B81] Kerkar S. P. (2013). Model T” cells: a time-tested vehicle for gene therapy. *Front. Immunol.* 4:304. 10.3389/fimmu.2013.00304 24098300 PMC3784795

[B82] Khuu H. M. Patel N. Carter C. S. Murray P. R. Read E. J. (2006). Sterility testing of cell therapy products: parallel comparison of automated methods with a CFR-compliant method. *Transfusion* 46 2071–2082. 10.1128/JCM.00302-09 17176318

[B83] Kikuchi T. Worgall S. Singh R. Moore M. A. Crystal R. G. (2000). Dendritic cells genetically modified to express CD40 ligand and pulsed with antigen can initiate antigen-specific humoral immunity independent of CD4+ T cells. *Nat. Med.* 6 1154–1159. 10.1038/80498 11017148

[B84] Kim D. Kim C. H. Moon J. I. Chung Y. G. Chang M. Y. Han B. S. (2009). Generation of human induced pluripotent stem cells by direct delivery of reprogramming proteins. *Cell Stem Cell* 4 472–476. 10.1016/j.stem.2009.05.005 19481515 PMC2705327

[B85] Kim K. Doi A. Wen B. Ng K. Zhao R. Cahan P. (2010). Epigenetic memory in induced pluripotent stem cells. *Nature* 467 285–290. 10.1038/nature09342 20644535 PMC3150836

[B86] Kimbrel E. A. Lanza R. (2015). Current status of pluripotent stem cells: moving the first therapies to the clinic. *Nat. Rev. Drug Discov.* 14 681–692. 10.1038/nrd4738 26391880

[B87] Koch C. M. Reck K. Shao K. Lin Q. Joussen S. Ziegler P. (2013). Pluripotent stem cells escape from senescence-associated DNA methylation changes. *Genome Res.* 23 248–259. 10.1101/gr.141945.112 23080539 PMC3561866

[B88] Kochenderfer J. N. Rosenberg S. A. (2013). Treating B-cell cancer with T cells expressing anti-CD19 chimeric antigen receptors. *Nat. Rev. Clin. Oncol.* 10 267–276. 10.1038/nrclinonc.2013.46 23546520 PMC6322669

[B89] Kochenderfer J. N. Wilson W. H. Janik J. E. Dudley M. E. Stetler-Stevenson M. Feldman S. A. (2010). Eradication of B-lineage cells and regression of lymphoma in a patient treated with autologous T cells genetically engineered to recognize CD19. *Blood* 116 4099–4102. 10.1182/blood-2010-04-281931 20668228 PMC2993617

[B90] Krawetz R. Taiani J. T. Liu S. Meng G. Li X. Kallos M. S. (2010). Large-scale expansion of pluripotent human embryonic stem cells in stirred-suspension bioreactors. *Tissue Eng. Part C Methods* 16 573–582. 10.1089/ten.TEC.2009.0228 19737071

[B91] Kropp C. Kempf H. Halloin C. Robles-Diaz D. Franke A. Scheper T. (2016). Impact of feeding strategies on the scalable expansion of human pluripotent stem cells in single-use stirred tank bioreactors. *Stem Cells Transl. Med.* 5 1289–1301. 10.5966/sctm.2015-0253 27369897 PMC5031176

[B92] Kropp C. Massai D. Zweigerdt R. (2017). Progress and challenges in large-scale expansion of human pluripotent stem cells. *Process Biochem.* 59 244–254. 10.1016/j.procbio.2016.09.032

[B93] Kurata H. Takakuwa K. Tanaka K. (1994). Vitrification of hematopoietic progenitor cells obtained from human cord blood. *Bone Marrow Transplant.* 14 261–263. 7994242

[B94] Kwok C. Ueda Y. Kadari A. Günther K. Ergün S. Heron A. (2018). Scalable stirred suspension culture for the generation of billions of human induced pluripotent stem cells using single-use bioreactors. *J. Tissue Eng. Regen. Med.* 12 e1076–e1087. 10.1002/term.2435 28382727

[B95] Lai R. C. Chen T. S. Lim S. K. (2011). Mesenchymal stem cell exosome: a novel stem cell-based therapy for cardiovascular disease. *Regen. Med.* 6 481–492. 10.2217/rme.11.35 21749206

[B96] Lai T. Yang Y. Ng S. K. (2013). Advances in mammalian cell line development technologies for recombinant protein production. *Pharmaceuticals* 6 579–603. 10.3390/ph6050579 24276168 PMC3817724

[B97] Lam A. T. Chen A. K. Li J. Birch W. R. Reuveny S. Oh S. K. (2014). Conjoint propagation and differentiation of human embryonic stem cells to cardiomyocytes in a defined microcarrier spinner culture. *Stem Cell Res. Ther.* 5:110. 10.1186/scrt498 25223792 PMC4183116

[B98] Larijani M. R. Seifinejad A. Pournasr B. Hajihoseini V. Hassani S. N. Totonchi M. (2011). Long-term maintenance of undifferentiated human embryonic and induced pluripotent stem cells in suspension. *Stem Cells Dev.* 20 1911–1923. 10.1089/scd.2010.0517 21198400

[B99] Lawson T. Kehoe D. E. Schnitzler A. C. Rapiejko P. J. Der K. A. Philbrick K. (2016). Process development for expansion of human mesenchymal stromal cells in a 50L single-use stirred tank bioreactor. *Biochem. Eng. J.* 120 49–62. 10.1016/j.bej.2016.11.020

[B100] Lebkowski J. (2011). GRNOPC1: the world’s first embryonic stem cell-derived therapy. *Regen. Med.* 6 11–13. 10.2217/rme.11.77 21999256

[B101] Lechanteur C. Baila S. Janssens M. E. Giet O. Briquet A. Baudoux E. (2014). Large-scale clinical expansion of mesenchymal stem cells in the GMP-compliant, closed automated Quantum^®^ cell expansion system: comparison with expansion in traditional t-flasks. *J. Stem Cell Res. Ther.* 4:1e11 10.4172/2157-7633.1000222

[B102] Liu P. Chen M. Liu Y. Qi L. S. Ding S. (2018). CRISPR-Based Chromatin Remodeling of the Endogenous Oct4 or Sox2 Locus Enables Reprogramming to Pluripotency. *Cell Stem Cell* 22 252–261. 10.1016/j.stem.2017.12.001 29358044

[B103] Lo B. Parham L. (2009). Ethical issues in stem cell research. *Endocr. Rev.* 30 204–213. 10.1210/er.2008-0031 19366754 PMC2726839

[B104] Löhle M. Hermann A. Glass A. Glaß H. Kempe A. Schwarz S. C. (2012). Differentiation efficiency of induced pluripotent stem cells depends on the number of reprogramming factors. *Stem Cells* 30 570–579. 10.1002/stem.1016 22213586

[B105] Lombardo A. Genovese P. Beausejour C. M. Colleoni S. Lee Y. L. Kim K. A. (2007). Gene editing in human stem cells using zinc finger nucleases and integrase-defective lentiviral vector delivery. *Nat. Biotechnol.* 25 1298–1306. 10.1038/nbt1353 17965707

[B106] Lou G. Chen Z. Zheng M. Liu Y. (2017). Mesenchymal stem cell-derived exosomes as a new therapeutic strategy for liver diseases. *Exp. Mol. Med.* 49 e346. 10.1038/emm.2017.63 28620221 PMC5519012

[B107] Mandai M. Watanabe A. Kurimoto Y. Hirami Y. Morinaga C. Daimon T. (2017). Autologous induced stem-cell-derived retinal cells for macular degeneration. *N. Engl. J. Med.* 376 1038–1046. 10.1056/NEJMoa1608368 28296613

[B108] Martin M. J. Muotri A. Gage F. Varki A. (2005). Human embryonic stem cells express an immunogenic nonhuman sialic acid. *Nat. Med.* 11:nm1181. 10.1038/nm1181 15685172

[B109] Matsuura K. Wada M. Shimizu T. Haraguchi Y. Sato F. Sugiyama K. (2012). Creation of human cardiac cell sheets using pluripotent stem cells. *Biochem. Biophys. Res. Commun.* 425 321–327. 10.1016/j.bbrc.2012.07.089 22842572

[B110] Mazzini L. Fagioli F. Boccaletti R. Mareschi K. Oliveri G. Olivieri C. (2003). Stem cell therapy in amyotrophic lateral sclerosis: a methodological approach in humans. *Amyotroph. Lateral Scler. Other Motor Neuron Disord.* 4 158–161. 10.1080/14660820310014653 13129802

[B111] Meirelles L. Nardi N. B. (2003). Murine marrow-derived mesenchymal stem cell: isolation, in vitro expansion, and characterization. *Br. J. Haematol.* 123 702–711. 10.1046/j.1365-2141.2003.04669.x 14616976

[B112] Menasché P. Vanneaux V. Hagège A. Bel A. Cholley B. Cacciapuoti I. (2015). Human embryonic stem cell-derived cardiac progenitors for severe heart failure treatment: first clinical case report. *Eur. Heart J.* 36 2011–2017. 10.1093/eurheartj/ehv189 25990469

[B113] Meng G. Liu S. Poon A. Rancourt D. E. (2017). Optimizing human induced pluripotent stem cell expansion in stirred-suspension culture. *Stem Cells Dev.* 26 1804–1817. 10.1089/scd.2017.0090 29017378

[B114] Miyazaki T. Futaki S. Suemori H. Taniguchi Y. Yamada M. Kawasaki M. (2012). Laminin E8 fragments support efficient adhesion and expansion of dissociated human pluripotent stem cells. *Nat. Commun.* 3:1236. 10.1038/ncomms2231 23212365 PMC3535336

[B115] Moon J. H. Lee J. R. Jee B. C. Suh C. S. Kim S. H. Lim H. J. (2008). Successful vitrification of human amnion-derived mesenchymal stem cells. *Hum. Reprod.* 23 1760–1770. 10.1093/humrep/den202 18541648

[B116] Mullard A. (2015). Novartis secures first CRISPR pharma collaborations. *Nat. Rev. Drug Discov.* 14:82 10.1038/nrd4546

[B117] Nakagawa M. Koyanagi M. Tanabe K. Takahashi K. Ichisaka T. Aoi T. (2008). Generation of induced pluripotent stem cells without Myc from mouse and human fibroblasts. *Nat. Biotechnol.* 26 101–106. 10.1038/nbt1374 18059259

[B118] Nakagawa M. Takizawa N. Narita M. Ichisaka T. Yamanaka S. (2010). Promotion of direct reprogramming by transformation-deficient Myc. *Proc. Natl. Acad. Sci. U.S.A.* 107 14152–14157. 10.1073/pnas.1009374107 20660764 PMC2922531

[B119] Nakagawa M. Tanabe K. Tezuka K. Shibata T. Kunisada T. Takahashi M. (2011). A more efficient method to generate integration-free human iPS cells. *Nat. Methods* 8 409–412. 10.1038/nmeth.1591 21460823

[B120] Naldini L. (2011). Ex vivo gene transfer and correction for cell-based therapies. *Nat. Rev. Genet.* 12 301–315. 10.1038/nrg2985 21445084

[B121] Nath S. C. Horie M. Nagamori E. Kino-Oka M. (2017). Size- and time-dependent growth properties of human induced pluripotent stem cells in the culture of single aggregate. *J. Biosci. Bioeng.* 124 469–475. 10.1016/j.jbiosc.2017.05.006 28601606

[B122] Nath S. C. Tokura T. Kim M. H. Kino-Oka M. (2018). Botulinum hemagglutinin-mediated in situ break-up of human induced pluripotent stem cell aggregates for high-density suspension culture. *Biotechnol. Bioeng.* 115 910–920. 10.1002/bit.26526 29278408

[B123] NHLBI (2009). *Urinary Exosome Protein Database. NHLBI. 2009-05-12. Retrieved 2009-10-11.* Bethesda, MD: NHLBI.

[B124] Oh S. K. Chen A. K. Mok Y. Chen X. Lim U. M. Chin A. (2009). Long-term microcarrier suspension cultures of human embryonic stem cells. *Stem Cell Res.* 2 219–230. 10.1016/j.scr.2009.02.005 19393590

[B125] Olmer R. Lange A. Selzer S. Kasper C. Haverich A. Martin U. (2012). Suspension culture of human pluripotent stem cells in controlled, stirred bioreactors. *Tissue Eng. Part C* 18 772–784. 10.1089/ten.TEC.2011.0717 22519745 PMC3460618

[B126] Orozco L. Munar A. Soler R. Alberca M. Soler F. Huguet M. (2013). Treatment of knee osteoarthritis with autologous mesenchymal stem cells: a pilot study. *Transplantation* 95 1535–1541. 10.1097/TP.0b013e318291a2da 23680930

[B127] Otsuji T. G. Bin J. Yoshimura A. Tomura M. Tateyama D. Minami I. (2014). A 3D sphere culture system containing functional polymers for large-scale human pluripotent stem cell production. *Stem Cell Rep.* 2:746. 10.1016/j.stemcr.2014.04.013 28081436 PMC4050484

[B128] Overton T. W. (2014). Recombinant protein production in bacterial hosts. *Drug Discov. Today* 19 590–601. 10.1016/j.drudis.2013.11.008 24246684

[B129] Pampusch M. S. Haran K. P. Hart G. T. Rakasz E. G. Rendahl A. K. Berger E. A. (2020). Rapid transduction and expansion of transduced T cells with maintenance of central memory populations. *Methods Clin. Dev. Protoc.* 16 1–10. 10.1016/j.omtm.2019.09.007 31673565 PMC6816036

[B130] Park Y. Chen Y. Ordovas L. Verfaillie C. M. (2014). Hepatic differentiation of human embryonic stem cells on microcarriers. *J. Biotechnol.* 174 39–48. 10.1016/j.jbiotec.2014.01.025 24480567

[B131] Pisitkun T. Shen R. F. Knepper M. A. (2004). Identification and proteomic profiling of exosomes in human urine. *Proc. Natl. Acad. Sci. U.S.A.* 101 13368–13373. 10.1073/pnas.0403453101 15326289 PMC516573

[B132] PR Newswire (2019). *Global Cell and Gene Therapy Market to Reach $11.96 Billion by 2025.* Available online at: https://www.prnewswire.com/news-releases/global-cell-and-gene-therapy-market-to-reach-11-96-billion-by-2025–300896848.html (accessed August 06, 2019).

[B133] Provasi E. Genovese P. Lombardo A. Magnani Z. Liu P. Q. Reik A. (2012). Editing T cell specificity towards leukemia by zinc finger nucleases and lentiviral gene transfer. *Nat. Med.* 18 807–815. 10.1038/nm.2700 22466705 PMC5019824

[B134] PRWeb (2018). Available online at: http://www.prweb.com/releases/2018/05/prweb15476972 (accessed April, 2020).

[B135] Rao M. S. Pei Y. Garcia T. Y. Chew S. Kasai T. Hisai T. (2018). Illustrating the potency of current Good Manufacturing Practice-compliant induced pluripotent stem cell lines as a source of multiple cell lineages using standardized protocols. *Cytotherapy* 20 861–872. 10.1016/j.jcyt.2018.03.037 29793831

[B136] Rayment E. A. Williams D. J. (2010). Concise review: mind the gap: challenges in characterizing and quantifying cell- and tissue-based therapies for clinical translation. *Stem Cells* 28 996–1004. 10.1002/stem.416 20333747 PMC2962908

[B137] Reardon S. Cyranoski D. (2014). Japan stem-cell trial stirs envy: researchers elsewhere can’t wait to test iPS cells in humans. *Nature* 513 278–288. 10.1038/513287a 25230622

[B138] Rehakova D. Souralova T. Koutna I. (2020). Clinical-grade human pluripotent stem cells for cell therapy: characterization strategy. *Int. J. Mol. Sci.* 21:E2435. 10.3390/ijms21072435 32244538 PMC7177280

[B139] Reisman M. Adams K. T. (2014). Stem cell therapy: a look at current research, regulations, and remaining hurdles. *Pharm. Ther.* 39 846–847, 854–857. PMC4264671 25516694

[B140] Rodrigues C. A. V. Fernandes T. G. Diogo M. M. da Silva C. L. Cabral J. M. S. (2011). Stem cell cultivation in bioreactors. *Biotechnol. Adv.* 29 815–829. 10.1016/j.biotechadv.2011.06.009 21726624

[B141] Roh K. H. Nerem R. M. Roy K. (2016). Biomanufacturing of therapeutic cells: state of the art, current challenges, and future perspectives. *Annu. Rev. Chem. Biomol. Eng.* 7 455–478. 10.1146/annurev-chembioeng-080615-033559 27276552

[B142] Rohani L. Fabian C. Holland H. Naaldijk Y. Dressel R. Löffler-Wirth H. (2016). Generation of human induced pluripotent stem cells using non-synthetic mRNA. *Stem Cell Res.* 16 662–672. 10.1016/j.scr.2016.03.008 27064648

[B143] Rohani L. Johnson A. A. Arnold A. Stolzing A. (2014). The aging signature: a hallmark of induced pluripotent stem cells? *Aging Cell* 13 2–7. 10.1111/acel.12182 24256351 PMC4326871

[B144] Rohani L. Johnson A. A. Naghsh P. Rancourt D. E. Ulrich H. Holland H. (2018). Concise review: molecular cytogenetics and quality control: clinical guardians for pluripotent stem cells. *Stem Cells Transl. Med.* 7 867–875. 10.1002/sctm.18-0087 30218497 PMC6265634

[B145] Rungarunlert S. Ferreira J. N. Dinnyes A. (2016). Novel bioreactor platform for scalable cardiomyogenic differentiation from pluripotent stem cell-derived embryoid bodies. *Methods Mol. Biol.* 1502 169–179. 10.1007/7651_2016_341 27044041

[B146] Sakuma T. Barry M. A. Ikeda Y. (2012). Lentiviral vectors: basic to translational. *Biochem. J.* 443 603–618. 10.1042/BJ20120146 22507128

[B147] Samavarchi-Tehrani P. Golipour A. David L. Sung H. K. Beyer T. A. Datti A. (2010). Functional genomics reveals a BMP-driven mesenchymal-to-epithelial transition in the initiation of somatic cell reprogramming. *Cell Stem Cell* 7 64–77. 10.1016/j.stem.2010.04.01 20621051

[B148] Schaber S. D. Gerogiorgis D. I. Ramachandran R. Evans J. M. Barton P. I. Trout B. L. (2011). Economic analysis of integrated continuous and batch pharmaceutical manufacturing: a case study. *I&EC* 50 10083–10092. 10.1021/ie2006752

[B149] Schellenberg A. Hemeda H. Wagner W. (2013). Tracking of replicative senescence in mesenchymal stem cells by colony-forming unit frequency. *Methods Mol. Biol.* 976 143–154. 10.1007/978-1-62703-317-6_11 23400440

[B150] Schwartz S. D. Regillo C. D. Lam B. L. Eliott D. Rosenfeld P. J. Gregori N. Z. (2015). Human embryonic stem cell-derived retinal pigment epithelium in patients with age-related macular degeneration and Stargardt’s macular dystrophy: follow-up of two open-label phase 1/2 studies. *Lancet* 385 509–516. 10.1016/S0140-6736(14)61376-3 25458728

[B151] Sensebe L. Bourin P. Tarte K. (2011). Good manufacturing practices production of mesenchymal stem/stromal cells. *Hum. Gene Ther.* 22 19–26. 10.1089/hum.2010.197 21028982

[B152] Serra M. Brito C. Correia C. Alves P. M. (2012). Process engineering of human pluripotent stem cells for clinical application. *Trends Biotechnol.* 30 350–359. 10.1016/j.tibtech.2012.03.003 22541338

[B153] Shafa M. Day B. Yamashita A. Meng G. Liu S. Krawetz R. (2012). Derivation of iPSCs in stirred suspension bioreactors. *Nat. Methods* 9 465–466. 10.1038/nmeth.1973 22484846

[B154] Shafa M. Panchalingam K. M. Walsh T. Richardson T. Baghbaderani B. A. (2019). Computational fluid dynamics modeling, a novel, and effective approach for developing scalable cell therapy manufacturing processes. *Biotechnol. Bioeng.* 116 3228–3241. 10.1002/bit.27159 31483482 PMC6973104

[B155] Shekaran A. Lam A. Sim E. Jialing L. Jian L. Wen J. T. P. (2016). Biodegradable ECM-coated PCL microcarriers support scalable human early MSC expansion and *in vivo* bone formation. *Cytotherapy* 18 1332–1344. 10.1016/j.jcyt.2016.06.016 27503763

[B156] Silva M. M. Rodrigues A. F. Correia C. Sousa M. F. Q. Brito C. Coroadinha A. S. (2015). Robust expansion of human pluripotent stem cells: integration of bioprocess design with transcriptomic and metabolomic characterization. *Stem Cells Transl. Med.* 4 731–742. 10.5966/sctm.2014-0270 25979863 PMC4479622

[B157] Singh H. Figliola M. J. Dawson M. J. Olivares S. Zhang L. Yang G. (2013). Manufacture of clinical-grade CD19-specific T cells stably expressing chimeric antigen receptor using Sleeping Beauty system and artificial antigen presenting cells. *PLoS One* 8:e64138. 10.1371/journal.pone.0064138 23741305 PMC3669363

[B158] Singh H. Mok P. Balakrishnan T. Rahmat S. N. Zweigerdt R. (2010). Upscaling single cell-inoculated suspension culture of human embryonic stem cells. *Stem Cell Res.* 4 165–179. 10.1016/j.scr.2010.03.001 20363202

[B159] Somerville R. P. Devillier L. Parkhurst M. R. Rosenberg S. A. Dudley M. E. (2012). Clinical scale rapid expansion of lymphocytes for adoptive cell transfer therapy in the WAVE(R) bioreactor. *J. Transl. Med.* 10:69. 10.1186/1479-5876-10-69 22475724 PMC3402993

[B160] Sommer C. A. Sommer A. G. Longmire T. A. Christodoulou C. Thomas D. D. Gostissa M. (2010). Excision of reprogramming transgenes improves the differentiation potential of iPS cells generated with a single excisable vector. *Stem Cells* 28 64–74. 10.1002/stem.255 19904830 PMC4848036

[B161] Song X. T. (2013). Combination of virotherapy and T-cell therapy: arming oncolytic virus with T-cell engagers. *Discov. Med.* 16 261–266. 24333405

[B162] Steiner D. Khaner H. Cohen M. Even-Ram S. Gil Y. Itsykson P. (2010). Derivation, propagation and controlled differentiation of human embryonic stem cells in suspension. *Nat. Biotechnol.* 28 361–364. 10.1038/nbt.1616 20351691

[B163] Sterlling J. (2018). Available online at: https://www.genengnews.com/insights/scaling-up-cell-therapy-manufacturing/ (accessed April, 2020).

[B164] Takahashi K. Tanabe K. Ohnuki M. Narita M. Ichisaka T. Tomoda K. (2007). Induction of pluripotent stem cells from adult human fibroblasts by defined factors. *Cell* 131 861–872. 10.1016/j.cell.2007.11.019 18035408

[B165] Taylor C. J. Bolton E. M. Pocock S. Sharples L. D. Pedersen R. A. Bradley J. A. (2005). Banking on human embryonic stem cells: estimating the number of donor cell lines needed for HLA matching. *Lancet* 366 2019–2025. 10.1016/S0140-6736(05)67813-0 16338451

[B166] Tekoah Y. Shulman A. Kizhner T. Ruderfer I. Fux L. Nataf Y. (2015). Large-scale production of pharmaceutical proteins in plant cell culture-the Protalix experience. *Plant Biotechnol. J.* 13 1199–1208. 10.1111/pbi.12428 26102075

[B167] Terakura S. Yamamoto T. N. Gardner R. A. Turtle C. J. Jensen M. C. Riddell S. R. (2012). Generation of CD19-chimeric antigen receptor modified CD8+ T cells derived from virus-specific central memory T cells. *Blood* 119 72–82. 10.1182/blood-2011-07-366419 22031866 PMC3251238

[B168] Thirumala S. Goebel W. S. Woods E. J. (2009). Clinical grade adult stem cell banking. *Organogenesis* 5 143–154. 10.4161/org.5.3.98113 20046678 PMC2781095

[B169] Thomson J. A. Itskovitz-Eldor J. Shapiro S. S. Waknitz M. A. Swiergiel J. J. Marshall V. S. (1998). Embryonic stem cell lines derived from human blastocysts. *Science* 282 1145–1147. 10.1126/science.282.5391.1145 9804556

[B170] Ting S. Chen A. Reuveny S. Oh S. K. (2014). An intermittent rocking platform for integrated expansion and differentiation of human pluripotent stem cells to cardiomyocytes in suspended microcarrier cultures. *Stem Cell Res.* 13 202–213. 10.1016/j.scr.2014.06.002 25043964

[B171] Trounson A. McDonald C. (2015). Stem cell therapies in clinical trials: progress and challenges. *Cell Stem Cell* 17 11–22. 10.1016/j.stem.2015.06.007 26140604

[B172] U.S. Food and Drug Administration (2020). *Cellular & gene therapy guidances.* Available online at: https://www.fda.gov/vaccines-blood-biologics/biologics-guidances/cellular-gene-therapy-guidances (accessed April 5, 2020).

[B173] Ullah I. Subbarao R. B. Rho G. J. (2015). Human mesenchymal stem cells - current trends and future prospective. *Biosci. Rep.* 35:e00191. 10.1042/BSR20150025 25797907 PMC4413017

[B174] US FDA (2008). *US FDA Guidance for Industry: CGMP for Phase 1 Investigational Drugs Regulation (EU) No 536/2014, Article 61(1).* Available online at: https://www.fda.gov/downloads/drugs/guidances/ucm070273.pdf (accessed April 6, 2020).

[B175] Valadi H. Ekström K. Bossios A. Sjöstrand M. Lee J. J. Lötvall J. O. (2007). Exosome-mediated transfer of mRNAs and microRNAs is a novel mechanism of genetic exchange between cells. *Nat. Cell Biol.* 9 654–659. 10.1038/ncb1596 17486113

[B176] Vosough M. Omidinia E. Kadivar M. Shokrgozar M. A. Pournasr B. Aghdami N. (2013). Generation of functional hepatocyte-like cells from human pluripotent stem cells in a scalable suspension culture. *Stem Cells Dev.* 22 2693–2705. 10.1089/scd.2013.0088 23731381

[B177] Wang J. Hao J. Bai D. Gu Q. Han W. Wang L. (2015). Generation of clinical-grade human induced pluripotent stem cells in Xeno-free conditions. *Stem Cell Res. Ther.* 6:223. 10.1186/s13287-015-0206-y 26564165 PMC4643509

[B178] Wang X. Riviere I. (2015). Manufacture of tumor- and virus-specific T lymphocytes for adoptive cell therapies. *Cancer Gene Ther.* 22 85–94. 10.1038/cgt.2014.81 25721207 PMC4480367

[B179] Wang X. Rivièrea I. (2016). Clinical manufacturing of CAR T cells: foundation of a promising therapy. *Mol. Ther. Oncolyt.* 3:16015. 10.1038/mto.2016.15 27347557 PMC4909095

[B180] Wang Y. Cheng L. Gerecht S. (2014). Efficient and scalable expansion of human pluripotent stem cells under clinically compliant settings: a view in 2013. *Ann. Biomed. Eng.* 42 1357–1372. 10.1007/s10439-013-0921-4 24132657 PMC4436032

[B181] Wang Y. Chou B. K. Dowey S. He C. Gerecht S. Cheng L. (2013). Scalable expansion of human induced pluripotent stem cells in the defined xeno-free E8 medium under adherent and suspension culture conditions. *Stem Cell Res.* 11 1103–1116. 10.1016/j.scr.2013.07.011 23973800 PMC4628790

[B182] Warikoo V. (2011). *Feasibility Study to Integrate Perfusion Cell Culture Processes to Continuous Downstream Processing.* Anaheim, CA: American Chemical Society.

[B183] Warren L. Manos P. D. Ahfeldt T. Loh Y. H. Li H. Lau F. (2010). Highly efficient reprogramming to pluripotency and directed differentiation of human cells with synthetic modified mRNA. *Cell Stem Cell* 7 618–630. 10.1016/j.stem.2010.08.012 20888316 PMC3656821

[B184] Woltjen K. Michael I. P. Mohseni P. Desai R. Mileikovsky M. Hämäläinen R. (2009). piggyBac transposition reprograms fibroblasts to induced pluripotent stem cells. *Nature* 458 766–770. 10.1038/nature07863 19252478 PMC3758996

[B185] Wuchter P. Bieback K. Schrezenmeier H. Bornhäuser M. Müller L. P. Bönig H. (2015). Standardization of good manufacturing practice-compliant production of bone marrow-derived human mesenchymal stromal cells for immunotherapeutic applications. *Cytotherapy* 17 128–139. 10.1016/j.jcyt.2014.04.002 24856898

[B186] Yamashita A. Liu S. Woltjen K. Thomas B. Meng G. Hotta A. (2016). Cartilage tissue engineering identifies abnormal human induced pluripotent stem cells. *Nat. Sci. Rep.* 3:1978. 10.1038/srep01978 23760219 PMC3680803

[B187] Yan Y. Song L. Tsai A. C. Ma T. Li Y. (2016). Generation of neural progenitor spheres from human pluripotent stem cells in a suspension bioreactor. *Methods Mol. Biol.* 1502 119–128. 10.1007/7651_2015_310 26837215

[B188] Yáñez-Muñoz R. J. Balaggan K. S. MacNeil A. Howe S. J. Schmidt M. Smith A. J. (2006). Effective gene therapy with nonintegrating lentiviral vectors. *Nat. Med.* 12 348–353. 10.1038/nm1365 16491086

[B189] Yang H. S. Jeon O. Bhang S. H. Lee S. H. Kim B. S. (2010). Suspension culture of mammalian cells using thermosensitive microcarrier that allows cell detachment without proteolytic enzyme treatment. *Cell Transplant.* 19 1123–1132. 10.3727/096368910X516664 20719079

[B190] Yehezkel S. Rebibo-Sabbah A. Segev Y. Tzukerman M. Shaked R. Huber I. (2011). Reprogramming of telomeric regions during the generation of human induced pluripotent stem cells and subsequent differentiation into fibroblast-like derivatives. *Epigenetics* 6 63–75. 10.4161/epi.6.1.13390 20861676 PMC3052915

[B191] Yin K. Wang S. Zhao R. C. (2019). Exosomes from mesenchymal stem/stromal cells: a new therapeutic paradigm. *Biomark Res.* 7:8. 10.1186/s40364-019-0159-x 30992990 PMC6450000

[B192] Zeng X. (2007). Human embryonic stem cells: mechanisms to escape replicative senescence? *Stem Cell Rev.* 3 270–279. 10.1007/s12015-007-9005-x 18026912

[B193] Zhang B. Yin Y. Lai R. C. Tan S. S. Choo A. B. Lim S. K. (2013). Mesenchymal stem cells secrete immunologically active Exosomes. *Stem Cells Dev.* 23 1233–1244. 10.1089/scd.2013.0479 24367916

[B194] Zimmermann A. Preynat-Seauve O. Tiercy J. M. Krause K. H. Villard J. (2012). Haplotype-based banking of human pluripotent stem cells for transplantation: potential and limitations. *Stem Cells Dev.* 21 2364–2373. 10.1089/scd.2012.0088 22559254

[B195] Zweigerdt R. Olmer R. Singh H. Haverich A. Martin U. (2011). Scalable expansion of human pluripotent stem cells in suspension culture. *Nat. Protoc.* 6 689–700. 10.1038/nprot.2011.318 21527925

